# Modelling the impact of changes in the extracellular environment on the cytosolic free NAD^+^/NADH ratio during cell culture

**DOI:** 10.1371/journal.pone.0207803

**Published:** 2018-11-29

**Authors:** Ross A. Kelly, Joseph Leedale, Andy Harrell, Daniel A. Beard, Laura E. Randle, Amy E. Chadwick, Steven D. Webb

**Affiliations:** 1 Department of Applied Mathematics, Liverpool John Moores University, Liverpool, United Kingdom; 2 EPSRC Liverpool Centre for Mathematics in Healthcare, Department of Mathematical Sciences, University of Liverpool, Liverpool, United Kingdom; 3 GlaxoSmithKline, David Jack Centre for Research, Ware, United Kingdom; 4 Department of Molecular & Integrative Physiology, University of Michigan, Ann Arbor, Michigan, United States of America; 5 Department of Pharmacy and Biomolecular Sciences, Liverpool John Moores University, Liverpool, United Kingdom; 6 MRC Centre for Drug Safety Science, Department of Molecular and Clinical Pharmacology, University of Liverpool, Liverpool, United Kingdom; Laurentian University, CANADA

## Abstract

Cancer cells depend on glucose metabolism via glycolysis as a primary energy source, despite the presence of oxygen and fully functioning mitochondria, in order to promote growth, proliferation and longevity. Glycolysis relies upon NAD^+^ to accept electrons in the glyceraldehyde-3-phosphate dehydrogenase (GAPDH) reaction, linking the redox state of the cytosolic NAD^+^ pool to glycolytic rate. The free cytosolic NAD^+^/NADH ratio is involved in over 700 oxidoreductive enzymatic reactions and as such, the NAD^+^/NADH ratio is regarded as a metabolic readout of overall cellular redox state. Many experimental techniques that monitor or measure total NAD^+^ and NADH are unable to distinguish between protein-bound and unbound forms. Yet total NAD^+^/NADH measurements yield little information, since it is the free forms of NAD^+^ and NADH that determine the kinetic and thermodynamic influence of redox potential on glycolytic rate. Indirect estimations of free NAD^+^/NADH are based on the lactate/pyruvate (L/P) ratio at chemical equilibrium, but these measurements are often undermined by high lability. To elucidate the sensitivity of the free NAD^+^/NADH ratio to changes in extracellular substrate, an *in silico* model of hepatocarcinoma glycolysis was constructed and validated against *in vitro* data. Model simulations reveal that over experimentally relevant concentrations, changes in extracellular glucose and lactate concentration during routine cancer cell culture can lead to significant deviations in the NAD^+^/NADH ratio. Based on the principles of chemical equilibrium, the model provides a platform from which experimentally challenging situations may be examined, suggesting that extracellular substrates play an important role in cellular redox and bioenergetic homeostasis.

## Introduction

Cellular bioenergetics describe the processes that generate energy in the form of ATP within the cell, achieved primarily via aerobic and anaerobic glycolysis, pyruvate and fatty acid oxidation, and oxidative phosphorylation within the mitochondria [1,2]. When glycolysis is coupled to oxidative phosphorylation, the NADH reducing equivalents produced in the cytosol by glyceraldehyde-3-phosphate dehydrogenase (GAPDH) are shuttled to the mitochondrial matrix where they are consumed by the respiratory chain. In the absence of oxygen, when mitochondria are unable to recycle the NADH to NAD^+^, lactate dehydrogenase provides an alternate means of oxidizing NADH to NAD^+^ to facilitate anaerobic glycolysis [3]. Tumours and highly-proliferating cells can show increased uptake of glucose, favouring glycolytic production of lactate, despite the presence of oxygen and fully functioning mitochondria [4]. The process of aerobic glycolysis under these conditions is also known as the Crabtree Effect, which is inefficient at producing ATP compared to the complete oxidation of glucose coupled to oxidative phosphorylation in terms of stoichiometric conversion of glucose to ATP [5]. However, the glycolytic rate can be considerably higher than oxidative respiration, and it has been suggested that this results in total ATP synthesis that is comparable over any given time, for either route of glucose metabolism [6]. Aerobic glycolysis in tumours and proliferating cells is recognised as an adaptive mechanism to facilitate rapid ATP production, aid survival and allow cells to thrive in the tumour microenvironment, and also to meet the elevated levels of biosynthesis required to support uncontrolled proliferation [7]. In turn, proliferating cells have a higher demand for reducing equivalents, in the form of NADH, which contributes to the NAD^+^/NADH ratio [8]. Therefore, an inextricable link exists between aerobic glycolytic rate and free NAD^+^/NADH redox state.

Current *in vitro* techniques for investigating the NAD^+^ pool redox state are only capable of measuring total NAD^+^ and NADH without discriminating between free and protein-bound forms [9]. This is problematic, as only free NAD^+^/NADH regulate cellular redox state, limiting the insight that can be gleaned from the total NAD^+^/NADH measurement. Free NAD^+^/NADH may be estimated or derived via: (i) exploitation of the lactate/pyruvate (L/P) ratio at equilibrium [10]; (ii) using hyperpolarised glucose [11]; or (iii) using genetically encoded sensors (SoNAR) [12]. Estimation of the free NAD^+^/NADH ratio using the L/P ratio is the most widely used approach, founded on the principles of chemical equilibrium, i.e., when the conversion between pyruvate + NADH and lactate + NAD^+^ is at equilibrium, the free NAD^+^/NADH ratio can be calculated by the following equation:

$$\frac{\left[NAD^{+}\right]}{\left[NADH\right]}=K_{eq}\times \frac{\left[pyruvate\right]}{\left[lactate\right]},$$ (1)

where,

$$K_{eq}=\frac{\left[pyruvate_{eq}][NADH_{eq}][H^{+}\right]}{\left[lactate_{eq}][NAD_{eq}^{+}\right]}=1.11\times 10^{-11}.$$ (2)

To use this method, the accurate definition of the equilibrium status for the conversion of the L/P ratio is crucial to correctly estimate the NAD^+^/NADH ratio [9]. Specifically, studies which assume the conversion is at equilibrium, whilst failing to confirm how close it is, can result in estimated ratios that are 1 to 2 orders of magnitude away from the true value. This is because the mass action reaction quotient (Q) at near equilibrium can differ between 1 and 2 orders of magnitude [13–19]. When Q = K_eq_, the forward and reverse rates of conversion are equal and there is no net loss or gain of lactate. However, when Q < K_eq_, the reaction favours formation of lactate and similarly, when Q > K_eq_, the reaction favours formation of pyruvate [9]. In cultured cancer cells, the conversion is predominantly from pyruvate to lactate, due to the rapid disposal of lactate via monocarboxylate transporters (MCTs) located on the plasma membrane [20,21]. This is a function of the high glycolytic rates found in cancer cells, which results in excessive production of pyruvate and NADH that is beyond the metabolic capacity of mitochondrial shuttles and pyruvate dehydrogenase [22]. To utilise the L/P ratio conversion to estimate free NAD^+^/NADH, one must force chemical equilibrium *in vitro* by elevating extracellular lactate concentrations between 16 and 22 mM [9]. However, in doing so, glucose consumption is seen to significantly reduce [9], thus demonstrating that the manipulation of extracellular lactate concentrations artificially alters the state of cellular lactate equilibrium while simultaneously perturbing glucose and energy metabolism.

One way of determining glycolytic rate *in vitro* is by extracellular flux analysis (EFA), which is relatively high throughput and experimentally inexpensive [23–25]. This method quantifies cellular respiration in the form of oxygen consumption rate (OCR) and extracellular acidification rate (ECAR). ECAR can be used as a measure of glycolytic rate when it is assumed that lactic acid, the terminal product of glycolysis, dissociates in the extracellular environment to a proton (H^+^) and lactate anion at physiological pH. Recently, the significance of respiratory contributions to ECAR has been highlighted, illustrating that the release of CO_2_ via oxidative phosphorylation can lead to the formation and dissociation of carbonic acid (H_2_CO_3_), amplifying and potentially distorting the ECAR output when used to assess glycolytic rate [26]. Fortunately, the proportion of respiratory acidification and glycolytic-only acidification can be easily calculated using the extracellular media buffering power (BP) [27]. This differentiation of acidification sources is necessary, as different cell types under different culture conditions may acidify the extracellular environment almost entirely via glycolysis or respiration [27]. Therefore, glycolytic proton production rate (PPR_gly_) is considered a more accurate representation of glycolytic rate compared to ECAR [27]. Current *in silico* models that focus specifically on hepatocellular bioenergetics in combination with EFA, are lacking in metabolic network depth and, as a result, authors tend to mathematically express glycolytic rate as pyruvate-to-lactate flux, rather than proton release into an extracellular environment, omitting respiratory contributions to ECAR altogether [27].

This study describes the construction, parameterisation and validation of an *in silico* model of hepatocarcinoma cell glycolysis used to investigate: (i) the sensitivity of the NAD^+^/NADH redox ratio to perturbations in extracellular lactate and glucose concentrations; and (ii) changes to GAPDH and LDH enzyme fluxes during variations in the extracellular substrate. The model described, captures the rapid binding and unbinding between protons and metal ions with all modelled biochemical species. This allows for the computation of the dynamic changes in pH from the total proton stoichiometry, which is crucial when simulating *in vitro* PPR_gly_ as a function of H^+^/lactate efflux into an extracellular environment. The model is validated against *in vitro* hepatocarcinoma EFA and NAD^+^-ATP data, to confirm the ability of the model to recreate the relationship between the two outputs. The HepG2 cell line was used due to the substantial number of studies that utilise these cells as a hepatic *in vitro* model for the study of bioenergetic toxicity [26,27]. Furthermore, this immortalized cell line was favoured over primary cells, as cancer cells are renowned for utilising the glycolytic pathway for the generation of cellular energy (ATP) over oxidative phosphorylation as a result of the Warburg effect, facilitating the study of ECAR as a function of glycolytic flux [5,27]. The two-point validation allows for changes in glycolytic rate as a function of NAD^+^/NADH perturbations to be explored. This approach aims to provide a platform from which aerobic glycolytic flux as a function of experimentally challenging situations may be investigated.

## Materials and methods

### In silico

#### Model development

**Fig 1:**
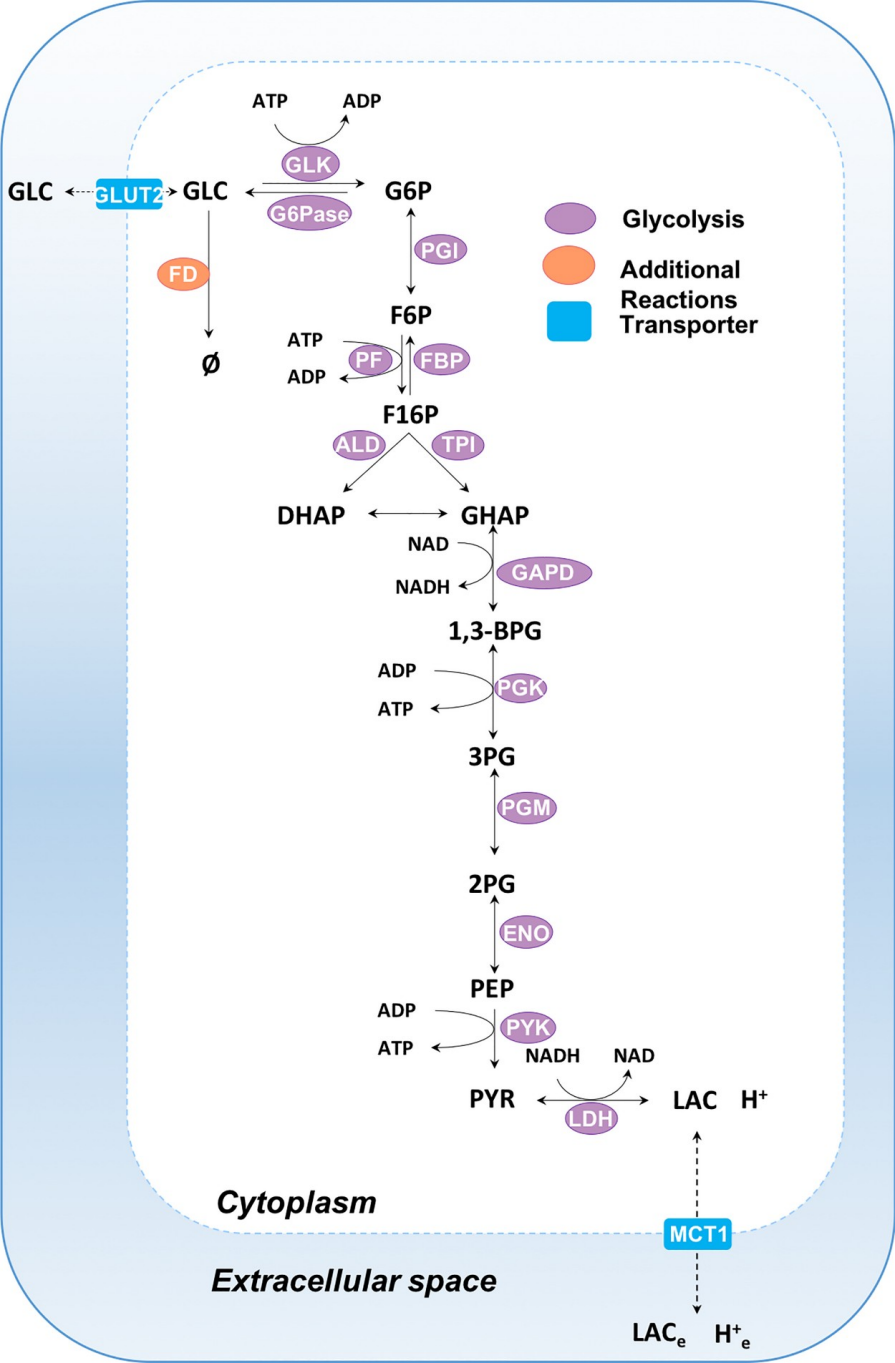
Schematic of the human hepatic bioenergetic model. The biochemical model comprises two compartments: cytoplasm and extracellular space. Glycolytic enzymes are shown in purple, transporter reactions are depicted as blue rectangles and additional reactions are portrayed in orange. Reaction descriptions can be found in Table 2. See S1 Supporting Information for additional information.

The mathematical model of hepatic glycolysis consists of 26 state variables, 14 enzyme-mediated reactions and two transport fluxes, occurring in two compartments: cytoplasm and extracellular space (Fig 1). Variable and reaction abbreviations are given in Tables 1 and 2 respectively. Reaction and transporter kinetics are modelled using kinetic terms and parameters sourced from the literature, or by fitting to experimental flux data. A comprehensive list of all kinetic terms can be found in the S1 Supporting Information. Flux units for the model are given as mM min^-1^.

**Table 1:** Model biochemical reactants including their corresponding abbreviations (Fig 1), allocated compartment and initial concentration.

Variable	Abbreviation	Compartment	Initial Concentration (mM)
Glucose	GLC	Cytoplasm	5.000
ATP	ATP	Cytoplasm	2.800
ADP	ADP	Cytoplasm	0.800
Glucose-6-phosphate	G6P	Cytoplasm	0.120
Fructose-6-phosphate	F6P	Cytoplasm	0.005
Inorganic phosphate	Pi	Cytoplasm	5.000
Fructose-1,6-phosphate	F16P	Cytoplasm	0.020
1,3-bisphospho-glycerate	BPG	Cytoplasm	0.300
Fructose-2,6-phosphate	F26P	Cytoplasm	0.004
Dihydroxyacetone-phosphate	DHAP	Cytoplasm	0.300
Glyceraldehyde-phosphate	GHAP	Cytoplasm	0.100
NAD	NAD	Cytoplasm	1.220
NADH	NADH	Cytoplasm	0.00056
2-phospho-D-glycerate	PG2	Cytoplasm	0.030
3-phospho-D-glycerate	PG3	Cytoplasm	0.270
Phosphoenolpyruvate	PEP	Cytoplasm	0.150
Pyruvate	PYR	Cytoplasm	0.100
Lactate	LAC	Cytoplasm	0.500
Protons	H	Cytoplasm	6.8 (pH)
Magnesium ions	Mg	Cytoplasm	5.000
Potassium ions	K	Cytoplasm	8.000
Glucose	GLC_e_	Extracellular	5.000
Lactate	LAC_e_	Extracellular	0.000
Protons	H_e_	Extracellular	7.4 (pH)
Magnesium ions	Mg_e_	Extracellular	0.000
Potassium ions	K_e_	Extracellular	0.000

Extracellular variables are distinguished from cytoplasm variables using subscript “e”.

**Table 2:** Model enzyme-mediated reactions including abbreviations and descriptions (Fig 1).

Reaction	Abbreviation	Description
Glucokinase	GLK	GLC + ATP → ADP + G6P + H
Glucose-6-phosphatase	G6Pase	G6P + H2O →G6P+ Pi
Phosphoglucose isomerase	PGI	G6P ⇋ F6P
Phosphofructokinase	PFK	F6P + ATP → F16P + ADP + H
Fructose-1,6-bisphosphatase	FBP1	F16P + H2O → F6P + Pi
Aldolase	ALD	F6P ⇋ DHAP + GAPH
Triosephosphate isomerase	TPI	DHAP ⇋ GAPH
Glyceraldehyde-3-Phosphate dehydrogenase	GAPDH	GAPH + Pi + NAD+⇋ BPG + NADH + H
Phosphoglycerate kinase	PGK	BPG + 2 ADP ⇋ PG3 + 2 ATP
Phosphoglycerate mutase 1	PGM	PG3 ⇋ PG2
Enolase / phosphopyruvate hydratase	ENO	PG2 ⇋ PEP
Pyruvate Kinase	PYK	PEP + 2 ADP + H ⇋ PYR + 2 ATP
Lactate dehydrogenase	LDH	PYR + NADH + H ⇋ LAC + NAD
Glucose Storage (Glycogenolysis)	FD	GLC → Ø
Glut-2-transporter	GLUT2	GLC_e_ ⇋ GLC
Monocarboxylate transporter 1	MCT1	LAC+ H⇋ LAC_e_ + H_e_

Single-headed reaction arrows indicate irreversible reactions and double arrows indicate reversible reactions. Full details of each reaction / transporter along with their corresponding parameter values can be found in the S1 Supporting Information.

### Modelling pH-dependent enzyme kinetics and reaction equilibria: BISEN

The hepatic glycolysis model was constructed in MATLAB, utilising the Biochemical Simulation Environment (BISEN) suite [28]. BISEN is an open-source tool that assists in generating sets of differential equations for simulating biochemical systems, accounting for dynamic proton and metal ion buffering, thermodynamics and reaction equilibria. Detailed instructions on how to use BISEN have been previously published [28]. Briefly, the state variables refer to the biochemical reactants within the model which are the sum of its interconvertible biochemical species. For example, ATP is a *reactant* that represents the sum of its related *species*: ATP^4-^, HATP^3-^, MgATP^2-^ etc. By accounting for the rapid interconversion of all species with metal ions and protons, the differences in state depending upon the pH can be modelled, whilst accounting for a complete proton stoichiometry. Each biochemical equation has its own associated equilibrium constant and the standard-state free energy of reaction, Δ_r_G^0^, that is independent of pH yet dependent upon changes in temperature and ionic strength. Overall, this allows favorability of a reaction to change as a result of a pH change [29].

### Kinetic equations and parameters

Transporter and glycolysis enzyme reaction equations, as well as initial parameter estimates, are all sourced from the literature (Table 2), predominantly from a comprehensive model of human hepatic glucose metabolism from Koenig *et al*. [30]. Additional reaction equations and parameters are based on experimental data from the literature or described here in this paper. All literature sourced parameter values can be found in the S1 Supporting Information. For model alignment with experimental data, certain model parameters were adjusted using unconstrained nonlinear optimization (Nelder-Mead simplex algorithm), starting with an initial literature-based parameter estimate. All rate equations and parameters are liver specific and can be found in the S1 Supporting Information along with their corresponding references.

### Model simulations

Model simulations were produced via integration of the resulting ordinary differential equations (ODEs) (S1 Supporting Information) using the variable order stiff solver ode15s (MATLAB). Compartment volumes were also set to mimic EFA by assigning the cytoplasm / intracellular volume as the volume occupied by the 2.5 × 10^4^ cells seeded per well, and the extracellular volume as 200 μl for the total extracellular volume of each well used in the EFA.

### *In silico*–*in vitro* PPR_gly_ coupling

*In silico* PPR_gly_ was simulated as a function of the MCT1 transport flux, *J*_*MCT*1_, expressed in mM min^-1^. Conversion from *in silico* transport flux of mM min^-1^ to the *in vitro* PPR_gly_ pmol min^-1^ / μg protein measurement was accomplished using Eq 3. Note, this conversion equation also includes normalization for experimental protein content, where *PNF* is the protein normalization factor.

$$PPR_{gly}=\frac{J_{MCT1}(2\times 10^{3})}{PNF}.$$ (3)

### In vitro

#### Materials

All extracellular flux analysis consumables were purchased from Seahorse Biosciences (North Billerica, MA, USA). HepG2 cells were purchased from the European Collection of Cell Cultures (ECACC, Salisbury, UK). Dulbecco’s modified media, Phosphate Buffered Saline (PBS) and Rat tail Collagen I were purchased from Life Technologies (Paisley, UK). All other reagents were purchased from Sigma Aldrich (Dorset, UK).

#### Cell culture

HepG2 cells were maintained in DMEM high-glucose media (glucose 25 mM) supplemented with foetal bovine serum (10% v/v), L-glutamine (2 mM), sodium pyruvate (1 mM) and HEPES (1 mM). Cells were incubated at 37°C under humidified air containing 5% CO_2_. Cells were used up to passage 17.

#### Extracellular flux analysis assay

HepG2 cells were collected on the day of the experiment by trypsinisation and then washed thrice with serum- and glucose-free media. The cells were then plated onto a collagen coated (50 μg/ml in acetic acid 0.02 M) XFe 96-well cell culture microplates (2.5 × 10^4^ cells/well) overnight in 100 μl of high glucose (25 mM) cell culture media. Before analysis, culture medium was removed from all wells and replaced with 175 μl of unbuffered glucose free Seahorse Assay media, supplemented with sodium pyruvate (1%v/v) and L-glutamate (1% v/v), pre-warmed to 37°C. Cells were then incubated in a CO_2_ free incubator at 37°C for 1 h. Before rate measurement, the XFe96 Instrument (Seahorse biosciences, North Billerica, MA) mixed the assay media in each well for 10 min, allowing the oxygen partial pressure to equilibrate. The oxygen consumption rate (OCR) and extracellular acidification rate (ECAR) were measured simultaneously thrice, establishing a baseline rate. For each measurement there was a 3 min mix followed by 3 min wait time to restore normal oxygen tension and pH in the transient microenvironment surrounding the cells. Glucose injections (0.1–25 mM) of 25 μl occurred at the end of the basal measurement cycles at 16 min, followed by 10 further measurements. The overall assay duration was 95 min for each of the n = 4 experimental repeats.

#### BCA protein quantification assay

Post extracellular flux analysis, assay medium was removed from all wells before the addition of 50 μl of Somatic cell ATP releasing agent (Sigma-Aldrich) to each well and the plate was carefully shaken (1 min, 300 RPM). A standard curve was prepared using a BCA stock (2 mg BCA/ml in ATP releasing agent). Working reagent (WR) was prepared by adding 50 parts bicinchoninic acid to 1 part copper sulphate. 5 μl of cell lysate was plated into a clear 96 well plate followed by addition of 200 μl of WR before incubation (37°C, for 30 min). The absorbance was then measured at 580 nm on a Labsystems Multiskan plate reader. Protein content was then extrapolated from the standard curve. Protein concentrations were then used to normalise the extracellular flux data, giving overall rates of ECAR and OCR as mpH min^-1^ well protein^-1^ and pmol min^-1^ well protein^-1^, respectively.

#### Buffering power

EFA assay media buffering capacity was measured at 37 ^o^C using a pH probe. Hydrochloric acid (HCl) (0.1 M) was charged in 6 x 20 μl aliquots to 10 ml of assay media, while changes in pH were recorded. Media buffering power was calculated from the gradient of the line of best fit after plotting the change in pH *vs* nmol H^+^ added per 2 μl [31].

#### PPR_gly_ calculations

PPR_gly_ was calculated from the ECAR measurements following the methodology of Mookerjee *et*. *al* (26,27). Briefly, ECAR was measured in units of mpH/min/well protein^-1^, representing respiratory and glycolytic contributions to acidification. The total proton production rate, PPR_tot_ (pmol H^+^/min/μg protein), was calculated using Eq (4).

$$PPR_{tot}=\frac{ECAR}{BP}.$$ (4)

The respiratory contributions to PPR, PPR_resp_ (pmol H^+^/min/μg protein), were calculated using Eq 5, where pK_1_ is the overall pKa for CO_2(aq)_ + H_2_O → HCO_3_^-^ = 6.093, max H^+^/O_2_ = 1 is the derived acidification for the metabolic transformation of glucose oxidation, the average total amount of oxygen consumption, denoted *OCR*_*tot*_, is equal to 17.78 pmol O_2_/min/μg protein for 5 mM over 10 measurements and non-respiratory oxygen consumption denoted *OCR*_*rot*_, is equal to 5.17 pmol O_2_/min/μg protein. Thus,

$$PPR_{resp}=\left(\frac{10^{pH-pK_{1}}}{1+10^{pH-pK_{1}}}\right)\left(\frac{\max H^{+}}{O_{2}}\right)(OCR_{tot}-OCR_{rot}).$$ (5)

Finally, using Eq (6), PPR_gly_ was calculated by subtracting respiratory acidification contributions from the total proton production rate:

$$PPR_{gly}=PPR_{tot}-PPR_{resp}.$$ (6)

### Statistical analysis

Statistical significance was ascertained using Prism 5 software via a one-way ANOVA, with values expressed as a mean ± standard deviation (S.D) taken from four independent experiments (n = 4 experimental repeats).

## Results and discussion

### Extracellular flux analysis

**Fig 2:**
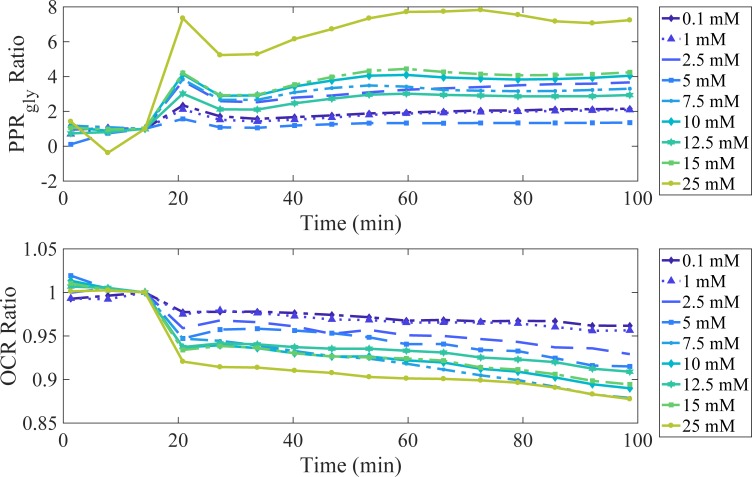
The effect of extracellular glucose on PPR_gly_ and OCR. HepG2 cells were exposed to serial concentrations of glucose (0.1 to 25 mM) at t = 16 minutes. PPR_gly_ and OCR values are normalised by the values obtained prior to glucose exposure and are expressed as the PPR_gly_ and OCR ratios compared to zero glucose added. Measurements are an average of n = 4 experimental repeats.

The effects of changes in extracellular glucose concentration (0–25 mM) on PPR_gly_ and OCR for HepG2 cells were examined (Fig 2).

Prior to EFA, the cells were starved for 60 minutes in glucose-free media. Glucose was reintroduced after 16 minutes of EFA, stimulating increases in PPR_gly_ ratio and decreases in OCR, for all concentrations of glucose (Fig 2). These results suggest that the introduction of extracellular glucose increases glycolytic energy metabolism, while diminishing respiratory energy production. An increase in glycolytic energy metabolism, facilitates ATP generation from glucose at a faster rate than oxidative phosphorylation. The ability of carcinoma cell lines, including HepG2, to exhibit this phenomena is well characterized and has been reported previously [4,32]. It is common practice to use high-glucose (25 mM) during routine cell culture. Fig 2 highlights how a high-glucose extracellular environment can influence cellular energy metabolism, illustrating that 25 mM glucose can yield up to a 7-fold increase in glycolytic-based energy metabolism when compared to a physiologically relevant extracellular glucose concentration (5 mM). In this instance, while glycolytic-based metabolism is primarily responsible for energy production, deducting respiratory contributions to extracellular acidification is an essential and facile endeavor for the sake of understanding the cellular bioenergetic output.

### Sensitivity analysis

Testing the sensitivity of a metabolic model with respect to its parameters is a crucial way of assessing its robustness. Variables that are most sensitive to parameter perturbation can be identified by measuring subsequent changes in time-course simulations and accordingly, any measurements or fluctuations in processes represented by these parameters must be carefully considered. Sensitivity analysis may be presented in many forms depending upon the state of the system. For this model, the relative change of the *j*^th^ variable with respect to a -99% to +400% change in the *i*^th^ parameter was measured (Eq 7). *V*_*j*_ is defined as the *j*^th^ variable over time. More specifically, $V_{j}^{ibase}$ is the *j*^th^ variable with its base value; $V_{j}^{ival}$ is the *j*^*th*^ variable with a perturbed value; with *ival ϵ* [-99,400%] of its base value, *ibase*. Mean *V*_*j*_(*t*) is denoted as $\bar{V_{j}}(t),$ the mean value of the *j*th variable over the time course *t ϵ* [0,300] min. Thus, our sensitivity metric, *X*, is defined:

$$X=\begin{array}{c} max\;variable\;change\\ relative\;to\\ parameter\;change \end{array}=\max\left(\frac{\left|\bar{V_{j}^{ival}}(t)-\bar{V_{j}^{ibase}}(t)\right|}{\bar{V_{j}^{ibase}}(t)}\frac{ibase}{\left|ival-ibase\right|}\right).$$ (7)

**Fig 3:**
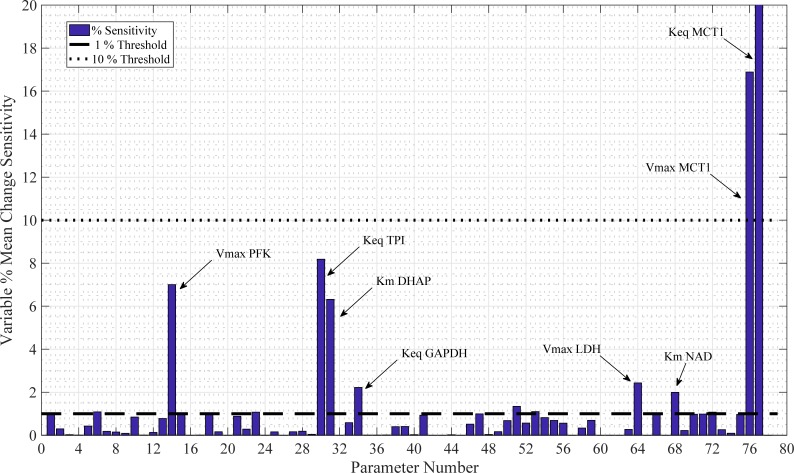
Sensitivity analysis. 78 model parameters were varied between -99% to +400% of their default values to identify the maximum mean change in any variable and provide a measure of sensitivity, *X*, relative to parameter change variation. The 8 most sensitive parameters are annotated.

**Fig 4:**
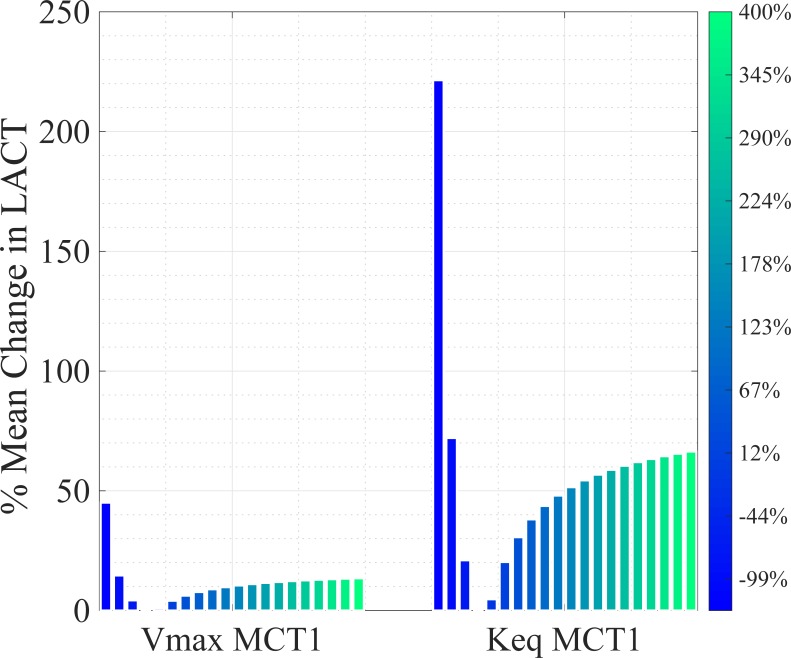
Sensitive model parameters illustrating changes in intracellular lactate concentration. % mean changes in intracellular lactate concentration as a function of sensitive parameter manipulation are shown compared to their initial steady state condition. Lactate is more susceptible to changes in the equilibrium constant, K_eq_, than to the V_max_ of MCT1 co-transporter kinetics.

A value of *X* = 1 would signify that the absolute, relative change in the mean of the variable over the time course is the same as the absolute, relative change in parameter. A parameter is classed as mildly sensitive (*MS*) if *X* is between 1 and 10. A parameter is sensitive (*S*) if *X* > 10. The sensitivity analysis results for the model are given in Fig 3. This analysis identified 8 key sensitive parameters: phosphofructokinase (PFK) V_max_ (*MS*); triosephosphate isomerase (TPI) K_eq_ (*MS*); dihydroxy-acetone phosphate (DHAP) K_m_ (*MS*); glyceraldehyde dehydrogenase (GAPDH) K_eq_ (*MS*); K_m_ NAD+ (*MS*); lactate dehydrogenase (LDH) V_max_ (*MS*); MCT1 K_eq_ (*S*); and MCT1 V_max_ (*S*). The two most sensitive parameters with respect to lactate, K_eq_ and V_max_ for the MCT1 transporter, are plotted as a % mean change of its initial value in Fig 4. With two out of three MCT1 transporter parameters registering as sensitive, parameter selection for this enzyme-mediated reaction must be carefully considered, especially when MCT1 transporter flux is to be used for simulating PPR_gly_.

Fig 4 shows the effects of changes in V_max_ MCT1 and K_eq_ MCT1 on intracellular lactate concentrations. The initial parameter was altered from -99% to +400% in 21 iterations, as shown with the 21 bars for each plot. Evidently, intracellular lactate concentration is more sensitive to the MCT1 equilibrium constant than the V_max_. However, both parameters satisfy the sensitive criteria threshold *X* > 10 (Fig 3).

### Model parameterisation: Cytoplasmic lactate content

**Fig 5:**
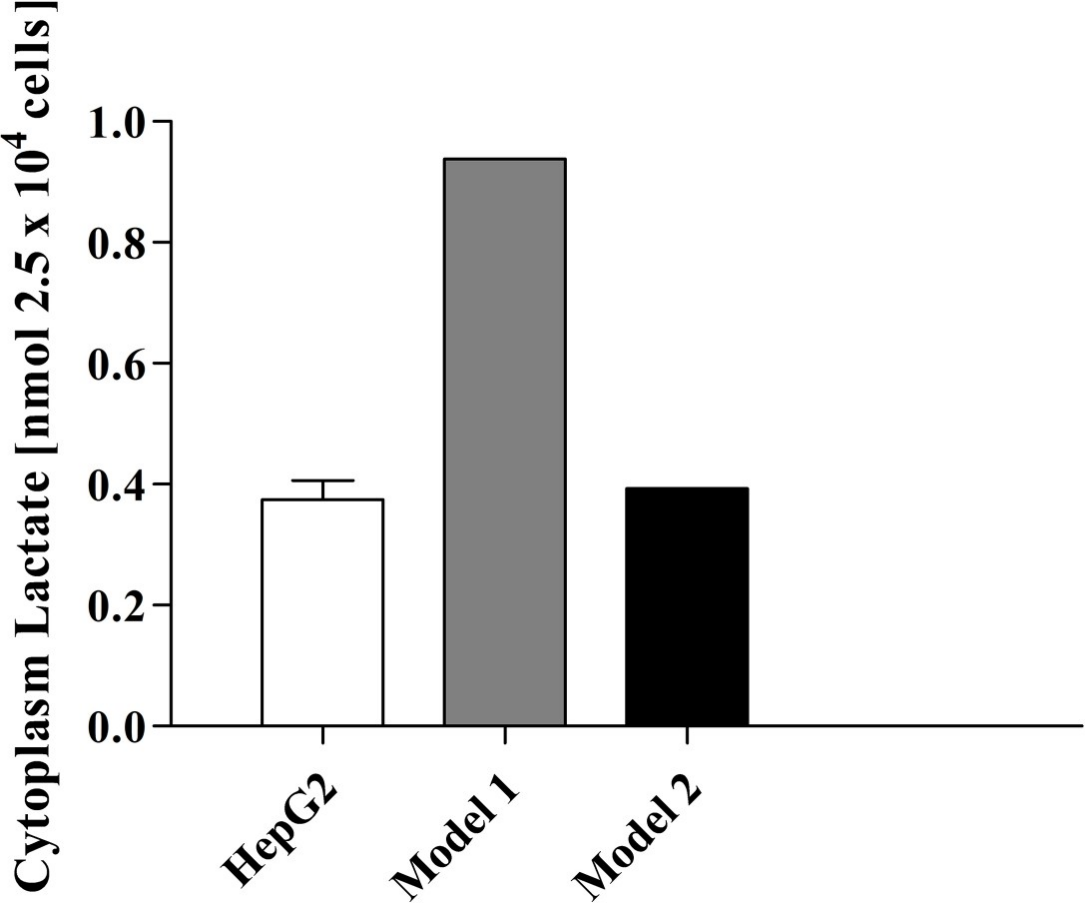
Intracellular lactate concentration after 48 h: Model intracellular lactate concentration was aligned with experimental data from HepG2 cells after 48 h. Model 1 simulation represents the lactate concentration pre-parameter adjustment with all model parameters obtained from the literature. Model 2 simulation represents post-parameter adjustment.

To accurately choose the values for the identified sensitive parameters, the model was fitted to *in vitro* intracellular lactate concentration. Liu *et al*. measured the intracellular lactate concentration of HepG2 cells during their study on the effects of miR-122 on pyruvate kinase [33]. Their data was used for comparison of the model simulations of the cytoplasm concentration of lactate over an extended time course of 48 h. Parameter adjustments were performed using unconstrained nonlinear optimization as described in the methods section, such that MCT1 V_max_ and MCT1 K_eq_ values were adjusted from 33 mM min^-1^ and 1, to 2.0×10^−3^ mM min^-1^ and 1.15×10^2^ respectively (equilibrium constants are unitless) in Fig 5. Steady state cytoplasmic levels of lactate in the model, prior to parameter adjustment, were approximately three times higher than *in vitro* amounts. A minor adjustment to the MCT1 V_max_ parameter provided a more comparable *in vitro–in silico* intracellular steady state concentration of lactate, while simultaneously leaving other variable and flux steady state concentrations largely unaltered.

### Model validation: Simulating EFA PPR_gly_ and the NAD^+^/ATP relationship

**Fig 6:**
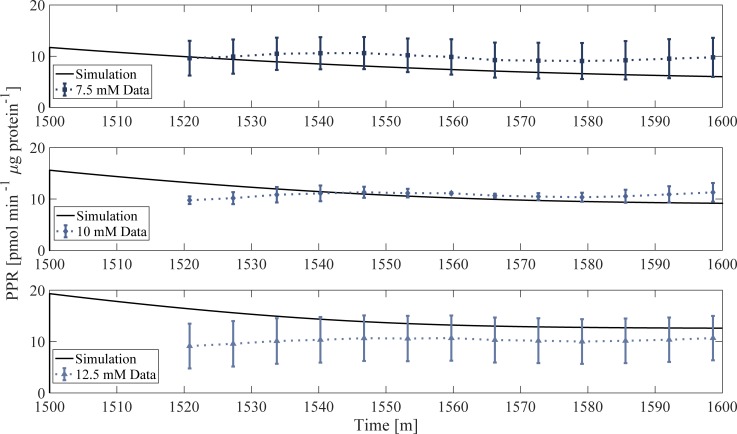
Model validation. Model simulation of PPR_gly_ (solid line) compared to *in vitro* PPR_gly_ data normalized to protein content. PPR_gly_ data was taken from EFA results (Fig 2) followed by adjusting each ECAR measurement for respiratory contributions. Prior to t = 1440 min, the model is simulated to steady state with extracellular glucose concentration of 25 mM (not shown). At t = 1440 min, extracellular glucose and lactate is removed to replicate *in vitro* procedure. At t = 1500 min, glucose is reintroduced at 5 mM allowing comparison to experimental data.

EFA PPR_gly_ experimental data not used for the original parameterisation was used to validate the model. The *in vitro* experiment consists of a 1440-min (24 h) incubation in a high glucose environment (25 mM), followed by extracellular lactate and glucose removal during a 60-min incubation in unbuffered media prior to EFA. Glucose is reintroduced at t = 1500 min after the end of the glucose-free incubation, followed by 80 min of measurements. The model replicated the EFA analysis data by generating the corresponding PPR_gly_ profile for 7.5, 10 and 12.5 mM of glucose, using the MCT1 flux term (Fig 6). The model output was normalized to the average protein content of the respective wells. The simulated PPR_gly_ is in good accordance with experimental observations, with the model being able to accurately simulate PPR_gly_ using the MCT1 flux only, suggesting that lactate/H^+^ is likely responsible for glycolytic extracellular acidification, which is in good agreement with the literature [34–36]. Furthermore, these simulations implement the cell incubation and media change features that occur prior to EFA, demonstrating the model’s ability to simulate extracellular changes that cannot be measured experimentally, and predict how the system responds to such perturbations. Simulation of the *in vitro* data inclusive of the incubation prior to EFA analysis provides confidence in the robustness of the model and its output with respect to glycolytic rate.

**Fig 7:**
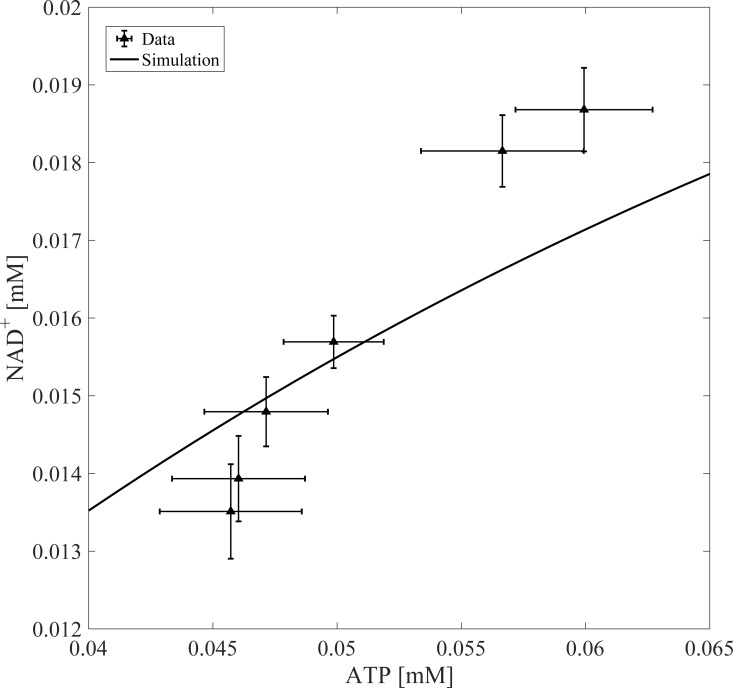
Model validation. Model output was validated by simulating changes in NAD^+^ as a function of ATP perturbations. The model was run to steady state before perturbing ATP concentrations. Experimental data was taken from Devin et. al [37].

Fig 7 shows further model validation by comparing *in silico* NAD^+^/ATP ratio outputs with experimental data [37]. NAD^+^ concentration in the cytoplasm depends on ATP concentration in liver cells such that linear increases in ATP lead to linear increases in NAD^+^ [37]. Model simulations mirror the experimentally observed positive correlation between ATP and NAD^+^. Note, the experimental data used in this section of model validation represents estimated NAD^+^ using the L/P derivation method, which may explain the slight discrepancy between the model output and the data. Overall, the model’s ability to simulate glycolytic rate, while capturing the essential dynamics between ATP and NAD^+^ concentration, demonstrates model fidelity with respect to the simulation of these two experimental outputs.

### Model predictions

#### NAD^+^/NADH redox state is sensitive to extracellular lactate and glucose concentration

**Fig 8:**
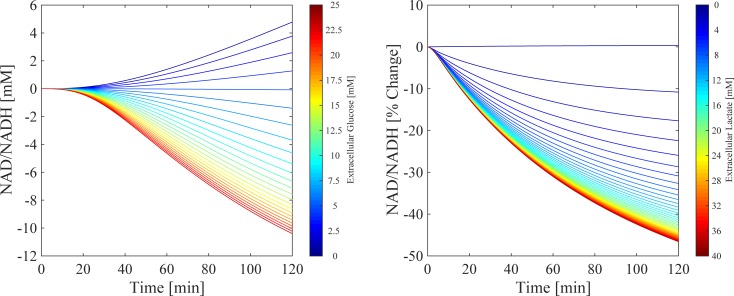
Extracellular substrate perturbations. Simulations of the resulting changes in NAD^+^/NADH ratio as a function of perturbations in extracellular glucose (A, 0 to 25mM) and lactate (B, 0 to 40 mM). Each simulation represents the percentage change in NAD^+^/NADH compared to an unperturbed simulation (black dashed line) over a 120-min period, with the perturbation of extracellular substrate occurring at 0 min. Time therefore represents time post-perturbation.

The model predictions for the sensitivity of the NAD^+^/NADH ratio during changes in extracellular substrates (glucose and lactate) are shown in Fig 8. Fig 8A shows the dynamic NAD^+^/NADH time-course profiles for an experimentally relevant range of extracellular glucose concentrations over a 120-min simulation. The model was first run to steady state (not shown), followed by perturbations from 0 to 25 mM of extracellular glucose. Each line represents the percentage change in the NAD^+^/NADH ratio compared to no change in extracellular glucose (black dashed line), where the initial conditions for the unperturbed simulations were 4.9 mM for extracellular glucose and 0.0012 mM for extracellular lactate. When extracellular glucose concentration is less than 5 mM, the model predicts a continuous increase in the NAD^+^/NADH ratio up to a maximum change of 4.8% at 120 min. Conversely, for concentrations of glucose greater than 5 mM, the model predicts a decrease in the NAD^+^/NADH ratio with a maximum decrease of 10.4% at 25 mM after 120 min. These model outputs suggest that, during hypoglycaemic conditions (< 5 mM), the model favours hepatic glucose production as opposed to utilisation, which would lead to a reduction in the concentration in NADH. During elevated glucose exposure (> 5 mM), the model output predicts increased glycolytic glucose utilisation, which is well documented experimentally (for every molecule of glucose metabolised via glycolysis, there is a net gain in 2 × NADH molecules) [38]. Therefore, as extracellular glucose concentrations increase, the glycolytic rate and NADH concentration increases, leading to a reduction in the NAD^+^/NADH ratio. The opposite is predicted when glucose is less than 5 mM.

Fig 8B illustrates the dynamic time-course outputs in NAD^+^/NADH during perturbations in extracellular lactate concentration from 0 to 40 mM over a 120-min simulation, representing the typical concentration range within a tumour microenvironment [39]. Following the initial steady state, each line represents the post-perturbation percentage change in the NAD^+^/NADH ratio compared to no change in extracellular lactate (black dashed line). As the concentration of extracellular lactate increases, the model predicts a continuous percentage decrease in the NAD^+^/NADH with a maximum decrease of 44.2% at 120 min. From a chemical equilibrium perspective, increases in extracellular lactate will promote uptake of lactate via the MCT1 which will in turn, alter Q to favour the conversion of lactate to pyruvate (when Q > K_eq_) [40]. An increase in the conversion of lactate to pyruvate means an increase in NADH and therefore a decrease in the NAD^+^/NADH ratio. Ultimately, simulations suggest that an increase in both extracellular substrates will favour the reduction of the NAD^+^/NADH ratio by increasing the concentration of NADH though induction of glycolysis during elevated extracellular glucose, and by alteration of the pyruvate to lactate conversion through Q during elevated extracellular lactate exposure.

#### Extracellular glucose and lactate influences NAD^+^/NADH through GAPDH and LDH fluxes

**Fig 9:**
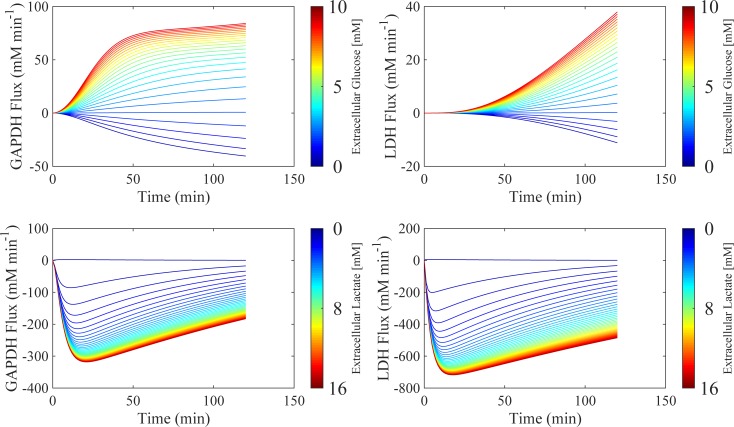
Extracellular substrate perturbations. Simulations of the resulting changes in GAPDH and LDH flux as a function of perturbations in extracellular glucose (0 to 25mM, top row panels) and lactate (0 to 40 mM, bottom row panels). Each simulation represents the percentage change in GAPDH and LDH flux compared to an unperturbed simulation (black dashed line) over a 120-min period, with the perturbation of extracellular substrate occurring at 0 min. Time therefore represents time post-perturbation.

Glycolytic regulation of cytosolic NAD^+^/NADH is maintained through two key enzymes: GAPDH and LDH. Perturbations in the reaction fluxes of these enzymes directly affects the NAD^+^/NADH ratio within the model. Therefore, the sensitivity of these key enzymes towards changes in the extracellular substrate environment was investigated. Fig 9 shows the resulting simulated changes in reaction fluxes for GAPDH and LDH during perturbations in extracellular glucose (0 to 25 mM, top panel) and extracellular lactate (0 to 40 mM, bottom panel) during a 120-minute simulation. Each line represents the percentage change in the enzyme flux compared to no change in extracellular glucose (black dashed line), using the same initial conditions as in Fig 8.

Simulations predict that for elevated extracellular glucose (> 5 mM), GAPDH and LDH enzyme fluxes increase up to a maximum of 84% and 37% respectively. During diminished extracellular glucose concentrations (< 5mM), the model predicts negative GAPDH and LDH fluxes, with a maximum decrease of -40% and -8.8% respectively. Negative reaction fluxes illustrate a switch in metabolic directionality whereby the reverse reaction governed by each enzyme is favoured. Consequently, when extracellular glucose concentration is high (> 5 mM), the model predicts increased formation of NADH through accelerated GAPDH flux, and increased NAD^+^ formation through accelerated LDH flux. While both NADH and NAD^+^ production is elevated, GAPDH is predicted to be more sensitive to extracellular glucose than LDH, with simulations suggesting that GAPDH flux is more than twice as fast as LDH. This results in a net increase in NADH, corresponding to the decreased NAD^+^/NADH ratio seen in Fig 8.

The bottom panel in Fig 9 shows how changes in extracellular lactate concentration affects GAPDH and LDH reaction flux. For all increased concentrations of extracellular lactate, simulations predict negative reaction fluxes for GAPDH, with a maximum change of 318% after 120 minutes. For the same changes in extracellular lactate concentration, the model also predicts a negative reaction flux for LDH, with a maximum of 716% over 120 minutes. Negative enzyme flux profiles suggest favouring of the production of NAD for GAPDH accompanied by the favouring of production of NADH for LDH. Overall, during increases in extracellular lactate concentration, the model predicts that there will again be a net increase in NADH concentration, given the differences in sensitivity between GAPDH and LDH.

It is unsurprising that increased extracellular lactate stimulates an increase in the reverse LDH flux, as tumour cells growing under aerobic conditions can utilise lactate as an energy source by uptake followed by conversion back to pyruvate, also known as metabolic symbiosis [39]. Lactate as an energy source in this instance spares glucose, making it more readily available for hypoxic tumour cells, with oxidative tumour cells preferring lactate as a source of metabolic fuel [41]. Furthermore, model predictions are in good accordance with the literature, which suggests that oxidation of lactate to pyruvate under these circumstances sustains NADH production in order to mitigate tumour oxidative stress [42]. While glycolytic regulation in general is complex, GAPDH is recognised as an important regulatory enzyme in living cancer cells, suggesting that GAPDH exhibits the most positive control on glycolytic flux according to metabolic control analysis [43]. GAPDH within the glycolytic pathway is regulated by ATP and NAD^+^, and with respect to aerobic glycolysis, GAPDH is highly expressed, emphasising its role in supporting the elevated demand for glycolysis [44]. Model simulations predict that the NAD^+^/NADH ratio may be manipulated through changes in GAPDH and LDH flux, as a function of perturbations in extracellular glucose and lactate.

### Model considerations and applications

The model presented here extends the mathematical/computational representation of *in vitro* EFA with regards to glycolytic rate. Other models such as the MITOsym, provide an outstanding computational representation of EFA, boasting inclusion of both oxidative and respiratory-based bioenergetic processes [45]. However, the MITOsym model is a significantly reduced representation of the bioenergetic portrait, aimed at capturing the key aspects of mitochondrial function in a whole cell environment [45]. In doing so, NADH is not explicitly modelled, but instead inferred from utilisation of pyruvate. Moreover, glycolysis itself is captured through a reduced set of ODEs, with ECAR computed using pyruvate-to-lactate flux (representative of LDH) [45]. Here, glycolytic rate is modelled using the glycolytic pathway in its entirety, including dynamic proton and metal ion buffering, thermodynamics and reaction equilibria. In doing so, the model is able to represent glycolytic rate specifically in the form that EFA measures it, i.e. PPR_gly_, using the MCT1 proton-lactate efflux process (Fig 6). Moreover, NAD^+^/NADH in this model is explicitly represented, capturing the relationship between NAD^+^ and ATP (Fig 7). Therefore, the model presented here is better suited for simulating and investigating glycolytic rate in the form of PPR_gly_. However, a caveat of this model is that it does not include TCA or oxidative metabolism and therefore its output is limited to represent non-oxidative energy metabolism only. Furthermore, while Ca^2+^ is present as a variable in the model, the omission of mitochondrial metabolism limits the models ability to explicitly capture other important regulators of the cytosolic NAD^+^/NADH ratio, for example, how the translation of cytosolic Ca^2+^ transients by the mitochondria results in transmission of NADH from the mitochondria itself to the cytosol [46]. The model predicts that the availability of extracellular substrates influences the NAD^+^/NADH ratio, particularly extracellular lactate (Fig 8). This is informative as *in vitro* derivation of the NAD^+^/NADH ratio by forcing chemical equilibrium using elevated concentrations of extracellular lactate could therefore lead to spurious estimations. Moreover, simulations predict that changes in GAPDH and LDH flux, as a function of perturbations in extracellular substrate, significantly influence glycolytic rate and the NAD^+^/NADH ratio (Fig 9). Indeed, the relationship between these enzymes and glycolytic rate is strengthened in Fig 3, where the parameters K_eq_ GAPDH and V_max_ LDH score as mildly sensitive with respect to PPR_gly_. The relationship between extracellular substrate and NAD^+^/NADH could be exploited, using the model to assist the design of experiments whereby extracellular substrates are deliberately manipulated to yield variance in the NAD^+^/NADH ratio. Such methods could perhaps be used to mimic inter-individual variation, metabolic disorders or cellular metabolic variations. At the very least, these simulations, which are in accordance with literature and *in vitro* outputs [10,41–43], suggest that the composition of extracellular substrates during cancer cell culture should be considered carefully due to their potential influence on the cellular free NAD^+^/NADH ratio and bioenergetic function.

## Conclusions

In this study, the sensitivity of the cytosolic free NAD^+^/NADH ratio towards perturbations in extracellular glucose and lactate was assessed using an *in silico* model of hepatocarcinoma glycolytic flux. The model predicts that the NAD^+^/NADH ratio is particularly sensitive to changes in extracellular lactate whereby elevated concentrations, comparable to those found in a tumour microenvironment, can result in a decrease in the NAD^+^/NADH ratio of up to 44.2% after 2 hours. The model was used to investigate how changes in extracellular glucose and lactate influence cancer bioenergetics through GAPDH and LDH flux, predicting that GAPDH and LDH are most sensitive to glucose and lactate respectively. Maximal changes in the enzyme fluxes of 318% and 716% for GAPDH and LDH are achieved, when extracellular glucose and lactate concentrations are 25 mM and 40 mM respectively. Overall, the model can be used to simulate experimentally challenging situations, such as circumventing the need to artificially alter the state of lactate equilibrium during estimation of the cytosolic free NAD^+^/NADH, while providing a platform from which experimental design of extracellular substrate manipulation can be assisted.

## Supporting information

S1 Supporting Information Model rate equations, kinetic parameters and statistical analysis. (DOCX)

S1 Data EFA normalised data. Zip file containing EFA data.
(ZIP)

S1 File Model MATLAB code. Zip file containing complete model code for simulation.
(ZIP)

S2 File MATLAB code html files. Zip file containing html versions of model code.
(ZIP)
