# Supplementary material for: Modelling the impact of changes in the extracellular environment on the cytosolic free NAD+/NADH ratio during cell culture
Source: PLoS One. 2018 Nov 29;13(11):e0207803. doi: 10.1371/journal.pone.0207803 (PMC6264472; doi:10.1371/journal.pone.0207803)
Supplement: S1 Supporting Information — (DOCX) [file pone.0207803.s001.docx]

**Modelling the impact of changes in the extracellular environment on the cytosolic free NAD^+^/NADH ratio during cell culture.**

**Ross A. Kelly^1^, Joseph Leedale^2*^, Andy Harrell^3^, Daniel A. Beard^4^, Laura E. Randle^5^, Amy E. Chadwick^6^ and Steve Webb^1^_._**

^1^ Department of Applied Mathematics, Liverpool John Moores University, Liverpool, UK.

^2^ EPSRC Liverpool Centre for Mathematics in Healthcare, Department of Mathematical Sciences, University of Liverpool, Liverpool, UK.

^3^ GlaxoSmithKline, David Jack Centre for Research, Ware, UK

^4^ Department of Molecular & Integrative Physiology, University of Michigan, Ann Arbor, Michigan, USA.

^5^ Department of Pharmacy and Biomolecular Sciences, Liverpool John Moores University, Liverpool, UK.

^6^ MRC Centre for Drug Safety Science, Department of Molecular and Clinical Pharmacology, University of Liverpool, Liverpool, UK.

**Supporting Information**

**S1. Supporting information contents**

| Appendix A | *In vitro* |
| --- | --- |
| Figure S1 | Glucose free EFA media buffering power |
| Table S1 | Model ODEs |
| Appendix B | *In silico* |
| Table S2 | Steady state variables vs literature range |
| Table S3 | Reaction & transport fluxes |
| Table S4 | Model ODEs |
| Appendix C | Enzyme & transporter kinetic terms and parameters values |
| Appendix D | *Statistical analysis* |
| Figure S2 | ECAR statistics report |
| Figure S3 | OCR statistics report |
| Figure S4 | Additional NAD^+^/NADH simulations |
| Figure S5 | *In vitro* EFA ECAR and OCR Data |

**S2. Description of Matlab Codes and Data files for simulating Fig 9**

| HGM.m | Driver program to simulate hepatocarcinoma glycolysis model |
| --- | --- |
| dXdT_HGM.m | Model ODE file |
| Plot_Fig9.m | File generating variable and flux steady state plots |
| setup.mat | Data file containing model parameters |
| x0b.mat | Data file containing variable initial conditions |
| html | html files of all code |

**Model construction**

The glycolytic flux model is based on the kinetic model of human hepatic glucose metabolism presented by Konig et al (33), gaining its pH-dependency and interwoven thermodynamics using the BISEN toolset (28). The model comprises of 14 enzyme mediated kinetic reactions and 2 transport fluxes between two compartments representing the cellular cytoplasm, where glycolysis is located, and the extracellular space. Compartment volumes are expressed as a ratio of total well volume. Compartment volumes and corresponding water fractions were set in the overall BSL construction file. In this model, the cytoplasm compartment represents the total intracellular volume occupied by 25E+03 cells, calculating using the density of liver tissue and radius of a HepG2 cell. Using a value of 9.0E-06 m for the cell radius r, the volume of 25E+03 cells V_1_, was calculated assuming a spherical cell.This yields a single cell intracellular volume of 3.0536E-15 m^3^.

The density of liver tissue is 1.077 g ml^-1^ and 1 m^-3^ = 1 L at this density (47). Using these values and scaling up from a single cell to 25E+03 cells therefore gives a total intracellular volume of 7.364E-08 L. The extracellular volume in this model is not the entire well volume but a microchamber volume. This is because EFA lowers a fluorescent measuring probe approximately 200 µm above the cell monolayer at the bottom of the well to measure OCR and ECAR creating a transient micro chamber. The extracellular compartment volume of the microchamber volume is 2.0E-06 L.

The compartments are then set as a fraction of the total volume. The total volume occupied by the cells and microchamber as a fraction of the total model volume is 0.0368/100 and 99.9632/100 respectively, with the total water fraction for both compartments being initially set to 1. Cytoplasm pH is set to a constant pH 6.8, as the source of intracellular proton production is omitted. Intracellular inorganic phosphate was held constant at 5 mM, in line with the Konig model (33). Intracellular free phosphate (Pi) is carefully maintained in cellular physiology by mechanisms not included within this model and was therefore held constant also. In the extracellular compartment, free magnesium and potassium ions were also held constant as the media used in EFA is an unbuffered solution.

**Appendix A**

**Figure S1: EFA media buffering power**

The buffering power of the unbuffered glucose-free extracellular flux analysis media was determined using a pH probe. The change in pH as a function of addition of hydrochloric acid (0.1 M) aliquots to 10 ml of media was measured. The change in pH was plotted against the amount of H^+^ per 7 µL charged.

|  |
| --- |
| **Figure S1. Glucose free EFA media buffering power.** The buffering power of unbuffered glucose-free EFA media was measured by adding 0.1 M HCl aliquots to 10 ml of media, followed by calculating the gradient of the slope from the resulting pH change. Data expressed as SD, with 3 experimental replicates. |

The media buffering power was calculated to be 0.8698 (mpH/pmol H^+^ 2 µl) for, used in the main paper to determine glycolytic acidification only for measurements devoid of glucose. For all measurements where glucose is present, the BP was set to 0.35 after scaling the literature value of 0.1 from 7 µl to 2 µl (26,27).

**Appendix B**

**Table S1: Model ODEs**

The model is constructed using enzyme mediated reaction kinetics, assembled together in the form of ordinary differential equations ODEs.

| \| **Variable** \| **Expression** \| \| --- \| --- \| \| *d*[GLC] */dt* = \| - *J*_GLK_ + *J*_G6PASE_ - *J*_FD_ + *J*_GLUT2_ \| \| *d*[ATP] */dt*  = \| - *J*_GLK_ - *J*_PFK_ + 2 *J*_PGK_ + 2 *J*_PYK_ \| \| *d*[ADP] */dt*  = \| *J*_GLK_ + *J*_PFK_ - 2 J_PGK_ - 2 J_PYK_ \| \| *d*[G6P] */dt*  = \| *J*_GLK_ - *J*_G6PASE_ - *J*_PGI_ \| \| *d*[F6P] */dt*  = \| *J*_PGI_ - *J*_PFK_ + *J*_FBP1_ \| \| *d*[F16P] */dt*  = \| *J*_PFK_ - *J*_FBP1_ - *J*_ALD_ \| \| *d*[BPG] */dt*  = \| *J*_GAPDH_ - *J*_PGK_ \| \| *d*[DHAP] */dt*  = \| *J*_ALD_ - *J*_TPI_ \| \| *d*[GHAP] */dt*  = \| *J*_ALD_ + *J*_TPI_ - *J*_GAPDH_ \| \| *d*[NAD] */dt*  = \| - *J*_GAPDH_ + *J*_LDH_ \| \| *d*[NADH] */dt*  = \| *J*_GAPDH_ - *J*_LDH_ \| \| *d*[PG2] */dt*  = \| *J*_PGK_ - *J*_PGYM_ \| \| *d*[PG3] */dt*  = \| *J*_PGYM_ - *J*_ENO_ \| \| *d*[PEP] */dt*  = \| *J*_ENO_ - *J*_PYK_ \| \| *d*[PYR] */dt*  = \| *J*_PYK_ - *J*_LDH_ \| \| *d*[LAC] */dt*  = \| *J*_LDH_ - *J*_LACT_ \| \| *d*[GLC_e] */dt*  = \| - *J*_GLUT2_ \| \| *d*[LAC_e] */dt*  = \| *J*_LACT_ \| |
| --- | --- | --- | --- | --- | --- | --- | --- | --- | --- | --- | --- | --- | --- | --- | --- | --- | --- | --- | --- | --- | --- | --- | --- | --- | --- | --- | --- | --- | --- | --- | --- | --- | --- | --- | --- | --- | --- | --- |
| **Table S1** Model biochemical reactants including their corresponding abbreviation (Figure 1), allocated compartment and initial concentration. Extracellular variables are distinguished from cytoplasm variables using subscript “e”. |

Model simulations were generated using Matlab 2017b, with absolute integration tolerance *absTol* = 10^-10^ and relative integration tolerance *relTol* = 10^-4^ with steady state solutions represented by changes in all concentrations less than the *absTol* for a timespan of ≥ 300 min. Complimentary variable time course solutions to main ECAR/PPR_gly_ simulations (Figure 7 main article) are shown in Figures S1 (variables *vs* time) and S2 (fluxes vs time). Extracellular variables are removed at t=20, with glucose reintroduced at 5 mM at t = 20 minutes, in order to replicate *in vitro* minor starvation prior to EFA.

**Table S2: Steady state simulations *vs* literature concentrations**

Model variables are expressed in mM, situated within either the cytoplasm or extracellular compartment. All initial concentrations were sourced from the literature (33).

| \| **Variable** \| **Model [mM]** \| **Literature [mM]** \| **Difference [mM]** \| \| --- \| --- \| --- \| --- \| \| GLC \| 4.7996 \| 5.0000 \| 0.2004 \| \| ATP \| 2.7203 \| 2.8000 \| 0.0797 \| \| ADP \| 0.8797 \| 0.8000 \| 0.0797 \| \| G6P \| 0.0336 \| 0.1200 \| 0.0864 \| \| F6P \| 0.0096 \| 0.0500 \| 0.0404 \| \| Pi \| 5.0000 \| 5.0000 \| 0.0000 \| \| F16P \| 0.3699 \| 0.0200 \| 0.3499 \| \| BPG \| 0.0040 \| 0.3000 \| 0.2960 \| \| F26P \| 0.4560 \| 0.0040 \| 0.4520 \| \| DHAP \| 0.0769 \| 0.0300 \| 0.0469 \| \| GHAP \| 1.0519 \| 0.1000 \| 0.9519 \| \| NAD \| 0.2049 \| 1.2200 \| 1.0151 \| \| NADH \| 0.1687 \| 0.0006 \| 0.1681 \| \| PG2 \| 0.4637 \| 0.0300 \| 0.4337 \| \| PG3 \| 0.0810 \| 0.2700 \| 0.1890 \| \| PEP \| 0.0956 \| 0.1500 \| 0.0544 \| \| PYR \| 0.1146 \| 0.1000 \| 0.0146 \| \| LAC \| 0.0054 \| 1.2000 \| 1.1946 \| |
| --- | --- | --- | --- | --- | --- | --- | --- | --- | --- | --- | --- | --- | --- | --- | --- | --- | --- | --- | --- | --- | --- | --- | --- | --- | --- | --- | --- | --- | --- | --- | --- | --- | --- | --- | --- | --- | --- | --- | --- | --- | --- | --- | --- | --- | --- | --- | --- | --- | --- | --- | --- | --- | --- | --- | --- | --- | --- | --- | --- | --- | --- | --- | --- | --- | --- | --- | --- | --- | --- | --- | --- | --- | --- | --- | --- | --- |
| **Table S2**: Literature and model variable concentration comparison. All literature values for variables are sourced from the liver specific glucose metabolism models (1, 4). |

**Appendix C: Flux Expressions & Corresponding Parameters**

All enzyme mediated/transporter reactions used in the model are addressed in this section, with its associated parameter values with it. Each reaction is illustrated, followed by the kinetic term, with parameters tabulated below. All parameter values are taken from the Konig model (33), with any adjusted parameters specified when appropriate.

## Glycolytic Flux Reactions

**Glucokinase (GLK):**


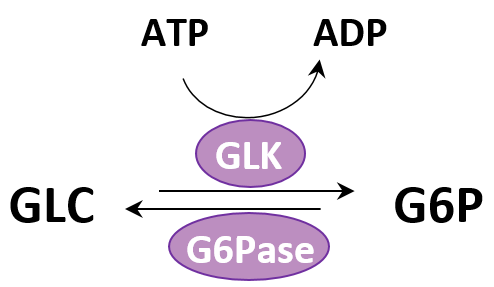


|  | $J_{GLK} =v_{max} {gk}_{free}\frac{[ATP]}{k_{m}^{ATP}+ [ATP]} \frac{{[GLC]}^{n}}{{[GLC]}^{n}+ \left( k_{m}^{glc} \right)^{n}} ,$ |  |
| --- | --- | --- |

where

|  | ${gk}_{free} =\frac{{[GLC]}^{n_{gkrp}}}{{[GLC]}^{n_{gkrp}}+ \left( k_{gkrp}^{GLC} \right)^{n_{gkrp}}} \left( 1- \frac{b_{gkrp}[F6P]}{\left[ F6P \right]+k_{gkrp}^{F6P}} \right) .$ |  |
| --- | --- | --- |

| **Parameter** | **Value** | **Units** |
| --- | --- | --- |
| *n* | 1.60×10^0^ |  |
| $k_{m}^{glc}$ | 7.50×10^0^ | mM |
| $k_{m}^{ATP}$ | 2.60×10^-1^ | mM |
| $n_{gkrp}$ | 2.00×10^0^ |  |
| $k_{gkrp}^{GLC}$ | 1.50×10^1^ | mM |
| $k_{gkrp}^{F6P}$ | 1.00×10^-2^ | mM |
| $b_{gkrp}$ | 7.00×10^-1^ |  |
| $v_{max}$ | 2.52×10^-2^ | mM min^-1^ |

Glucokinase (GLK) is regulated by the glucokinase regulator protein which is a competitive inhibitor for glucose. The regulatory binding mechanism of this protein is dependent upon glucose and fructose-6-phosphate (F6P) which in turn alters the k_m_ for glucose.

**Glucose-6-phosphatase (G6Pase):**


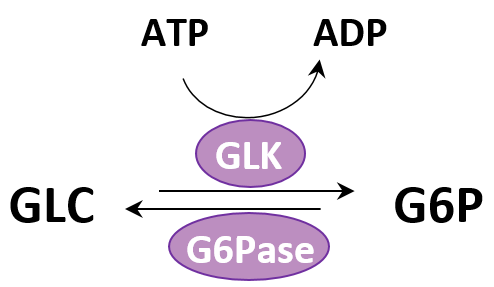


|  | $J_{G6PASE} =v_{max}\frac{\left[ G6P \right]}{k_{m}^{G6P}+\left[ G6P \right]} .$ |  |
| --- | --- | --- |

| **Parameter** | **Value** | **Units** |
| --- | --- | --- |
| $k_{m}^{G6P}$ | 2.00×10^0^ | mM |
| $v_{max}$ | 1.89×10^-2^ | mM min^-1^ |

**Glucose-6-phosphate isomerase (PGI):**


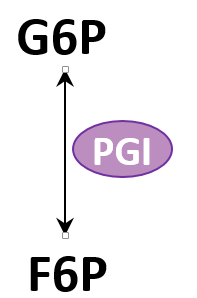


|  | $J_{PGI} =\frac{\frac{v_{max}}{k_{m}^{G6P}} \left( \left[ G6P \right]-\frac{\left[ F6P \right]}{K_{eq}} \right)}{1+ \frac{\left[ G6P \right]}{k_{m}^{G6P}}+\frac{\left[ F6P \right]}{k_{m}^{F6P}}} .$ |  |
| --- | --- | --- |

| **Parameter** | **Value** | **Units** |
| --- | --- | --- |
| $K_{eq}$ | 5.17×10^-1^ |  |
| $k_{m}^{G6P}$ | 1.82×10^-1^ | mM |
| $k_{m}^{F6P}$ | 7.10×10^-2^ | mM |
| $v_{max}$ | 4.20×10^-1^ | mM min^-1^ |

**Phosphofructokinase (PFK):**


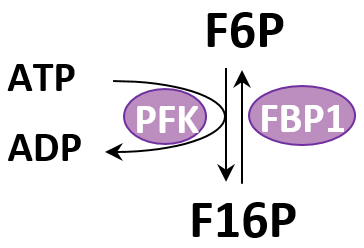


| $J_{PFK} =v_{max}\left( 1+\frac{\left[ F16P \right]}{k_{a}^{F16P}} \right)\left( \frac{\left[ G6P \right]\left[ ATP \right]}{k_{i}^{F16P}k_{m}^{ATP}+k_{m}^{F6P}\left[ ATP \right]+k_{m}^{ATP}\left[ F6P \right]+\left[ ATP \right]\left[ F6P \right]} \right) .$ |
| --- |

| **Parameter** | **Value** | **Units** |
| --- | --- | --- |
| $k_{m}^{ATP}$ | 1.11×10^-1^ | mM |
| $k_{m}^{F6P}$ | 7.70×10^-2^ | mM |
| $k_{i}^{F16P}$ | 1.20×10^-2^ | mM |
| $v_{max}$ | 7.18×10^-2^ | mM min^-1^ |
| $k_{a}^{F16P}$ | 1.00×10^-3^ | mM |

**Fructose-1,6-bisphosphatase (FBP):**


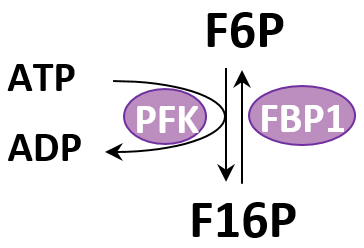


|  | $J_{FBP} =\frac{v_{max}}{1+\frac{\left[ F26P \right]}{k_{i}^{F26P}}}\left( \frac{\left[ F16P \right]}{{\left[ F16P \right]+k}_{m}^{F16P}} \right) .$ |  |
| --- | --- | --- |

| **Parameter** | **Value** | **Units** |
| --- | --- | --- |
| $k_{i}^{F26P}$ | 1.00×10^-3^ | mM |
| $k_{m}^{F16P}$ | 1.30×10^-3^ | mM |
| $v_{max}$ | 4.33×10^-1^ | mM min^-1^ |

**Aldolase (ALD):**


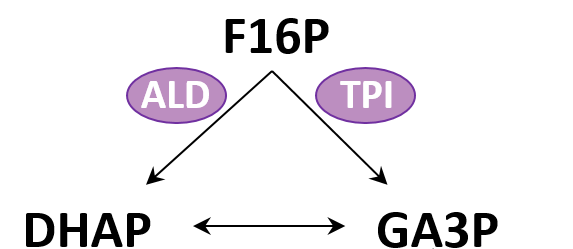


| $J_{ALD} =\frac{\frac{v_{max}}{k_{m}^{F16P}}\left( \left[ F16P \right]\frac{\left[ GAPDH \right]\left[ DHAP \right]}{K_{eq}} \right)}{\begin{aligned} 1+\frac{\left[ F16P \right]}{k_{m}^{F16P}}+\frac{\left[ GAPDH \right]}{k_{i}^{GAPDH}}+\frac{\left[ DHAP \right]\left( \left[ DHAP \right]+k_{m}^{GAPDH} \right)}{k_{m}^{DHAP}k_{i}^{GAPDH}}+\frac{\left[ F16P \right]\left[ GAPDH \right]}{k_{m}^{F16P}k_{i2}^{GRAP}} \end{aligned}} .$ |
| --- |

| **Parameter** | **Value** | **Units** |
| --- | --- | --- |
| $K_{eq}$ | 9.76×10^-5^ | mM |
| $k_{m}^{F16P}$ | 7.10×10^-3^ | mM |
| $v_{max}$ | 4.20×10^-3^ | mM min^-1^ |
| $k_{m}^{DHAP}$ | 3.64×10^-2^ | mM |
| $k_{m}^{GAPDH}$ | 7.10×10^-3^ | mM |
| $k_{i}^{GAPDH}$ | 5.72×10^-2^ | mM |
| $k_{i2}^{GRAP}$ | 4.2×10^-1^ | mM |

**Triosephosphate isomerase (TPI):**


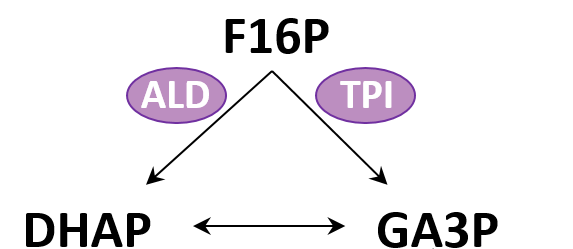


|  | $J_{TPI} =\frac{v_{max}}{k_{m}^{DHAP}}\left( \frac{\left[ DHAP \right]-\frac{\left[ GAPDH \right]}{K_{eq}}}{1+ \frac{\left[ DHAP \right]}{k_{m}^{DHAP}}+\frac{\left[ GAPDH \right]}{k_{m}^{GAPDH}}} \right) .$ |  |
| --- | --- | --- |

| **Parameter** | **Value** | **Units** |
| --- | --- | --- |
| $K_{eq}$ | 5.45×10^-2^ | mM |
| $k_{m}^{DHAP}$ | 5.90×10^-1^ | mM |
| $v_{max}$ | 4.20×10^-3^ | mM min^-1^ |
| $k_{m}^{GAPDH}$ | 4.20×10^-1^ | mM |

**Glyceraldehyde-3-phosphate dehydrogenase (GAPDH):**


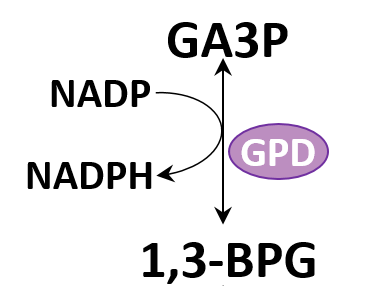


| $J_{GAPDH} =\frac{\frac{v_{max}}{k_{NAD}k_{GAPDH}k_{Pi}}\left( \left[ NAD \right]\left[ GAPDH \right]\left[ Pi \right]-\frac{\left[ BPG \right]\left[ NADH \right]}{K_{eq}} \right)}{\begin{aligned} \begin{aligned} \left( 1+\frac{\left[ NAD \right]}{k_{NAD}} \right)+ \end{aligned}\begin{aligned} \left( 1+\frac{\left[ GAPDH \right]}{k_{GAPDH}} \right)+\left( 1+\frac{\left[ Pi \right]}{k_{Pi}} \right) \end{aligned}\begin{aligned} +\left( 1+\frac{\left[ NADH \right]}{k_{NADH}} \right)\left( 1+\frac{\left[ BPG \right]}{k_{BPG}} \right)-1 \end{aligned} \end{aligned}} .$ |
| --- |

| **Parameter** | **Value** | **Units** |
| --- | --- | --- |
| $K_{eq}$ | 8.68×10^-2^ |  |
| $k_{NAD}$ | 5.00×10^-2^ | mM |
| $v_{max}$ | 4.20×10^-3^ | mM min^-1^ |
| $k_{m}^{GAPDH}$ | 5.00×10^-4^ | mM |
| $k_{Pi}$ | 3.90×10^0^ | mM |
| $k_{NADH}$ | 8.30×10^-3^ | mM |
| $k_{BPG}$ | 3.50×10^-12^ | mM |

**Phosphoglycerate Kinase (PGK):**


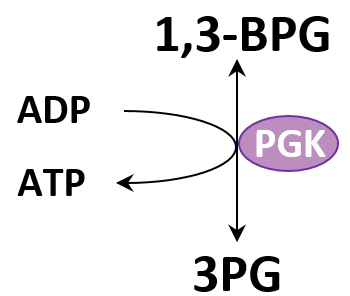


|  | $J_{PGK} =\frac{\frac{v_{max}}{k_{ADP}k_{BPG}}\left( \left[ ADP \right]\left[ BPG \right]-\frac{\left[ ATP \right]\left[ PG3 \right]}{K_{eq}} \right)}{\begin{aligned} \begin{aligned} \left( 1+\frac{\left[ ADP \right]}{k_{ADP}} \right)+ \end{aligned}\begin{aligned} \left( 1+\frac{\left[ BPG \right]}{k_{BPG}} \right)+\left( 1+\frac{\left[ ATP \right]}{k_{ATP}} \right) \end{aligned}\begin{aligned} +\left( 1+\frac{\left[ PG3 \right]}{k_{PG3}} \right)-1 \end{aligned} \end{aligned}} .$ |  |
| --- | --- | --- |

| **Parameter** | **Value** | **Units** |
| --- | --- | --- |
| $K_{eq}$ | 7.00×10^0^ |  |
| $k_{ADP}$ | 3.50×10^-1^ | mM |
| $v_{max}$ | 4.20×10^-3^ | mM min^-1^ |
| $k_{BPG}$ | 2.00×10^-3^ | mM |
| $k_{ATP}$ | 4.80×10^-1^ | mM |
| $k_{PG3}$ | 1.20×10^0^ | mM |

**Phosphoglycerate mutase (PGM):**


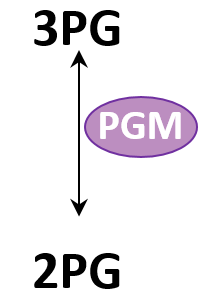


| $J_{PGM} =\frac{v_{max}\left( \left[ PG3 \right]-\frac{\left[ PG2 \right]}{K_{eq}} \right)}{\begin{aligned} \begin{aligned} \left[ PG3 \right]+k_{PG3} \end{aligned}\begin{aligned} \left( 1+\frac{\left[ PG2 \right]}{k_{PG2}} \right) \end{aligned} \end{aligned}} .$ |
| --- |

| **Parameter** | **Value** | **Units** |
| --- | --- | --- |
| $K_{eq}$ | 1.84×10^-1^ |  |
| $k_{PG3}$ | 5.00×10^0^ | mM |
| $v_{max}$ | 4.20×10^-3^ | mM min^-1^ |
| $k_{PG2}$ | 1.00×10^0^ | mM |

**Enolase (ENO):**


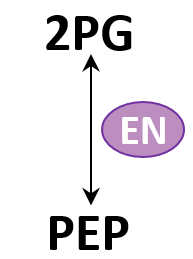


| $J_{ENO} =\frac{v_{max}\left( \left[ PG2 \right]-\frac{\left[ PEP \right]}{K_{eq}} \right)}{\begin{aligned} \begin{aligned} \left[ PG2 \right]+k_{PG2} \end{aligned}\begin{aligned} \left( 1+\frac{\left[ PEP \right]}{k_{PEP}} \right) \end{aligned} \end{aligned}} .$ |
| --- |

| **Parameter** | **Value** | **Units** |
| --- | --- | --- |
| $K_{eq}$ | 5.45×10^-1^ |  |
| $k_{PG2}$ | 1.00×10^0^ | mM |
| $v_{max}$ | 3.60×10^-3^ | mM min^-1^ |
| $k_{PEP}$ | 1.00×10^0^ | mM |

**Pyruvate Kinase (PK):**


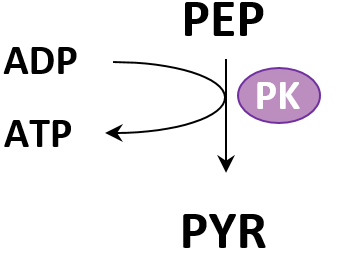

$$J_{PK}=\left( 1-\gamma\right)v^{dp}+\gamma v^{p} ,$$

where

$$v^{dp} =v_{max} \alpha_{inp}^{dp}\left( \frac{\left[ PEP \right]^{n^{PEP}}}{\left[ PEP \right]^{n^{PEP}}+\left( k_{pep,inp}^{dp} \right)^{n^{PEP}}} \right)\left( \frac{\left[ ADP \right]}{\left[ ADP \right]+k_{ADP}} \right) ,$$

and

$$v^{p} =v_{max} \alpha_{inp}^{p}\left( \frac{\left[ PEP \right]^{n^{PEP}}}{\left[ PEP \right]^{n^{PEP}}+\left( k_{pep,inp}^{p} \right)^{n^{PEP}}} \right)\left( \frac{\left[ ADP \right]}{\left[ ADP \right]+k_{ADP}} \right) ,$$

given

$$\left( \alpha_{base}^{p}+\left( 1-\alpha_{base}^{p} \right)f \right) ,$$

$$\left( \alpha_{base}^{dp}+\left( 1-\alpha_{base}^{dp} \right)f \right) ,$$

and

$$\alpha_{inp}^{dp} =\left( 1-f \right)\left( \alpha^{dp}-\alpha_{end} \right)+\alpha_{end},$$

$$\alpha_{inp}^{p} =\left( 1-f \right)\left( \alpha^{p}-\alpha_{end} \right)+\alpha_{end} ,$$

$$k_{pep,inp}^{dp} =\left( 1-f \right)\left( k_{pep}^{dp}-k_{pep}^{min} \right)+k_{pep}^{min} ,$$

$$k_{pep,inp}^{p} =\left( 1-f \right)\left( k_{pep}^{p}-k_{pep}^{min} \right)+k_{pep}^{min} ,$$

with

$$f=\frac{\left[ F16P \right]^{n_{fbp}}}{\left[ F16P \right]^{n_{fbp}}+\left( k_{fbp}^{dp} \right)^{n_{fbp}}}.$$

| **Parameter** | **Value** | **Units** |
| --- | --- | --- |
| $n^{PEP}$ | 3.50×10^0^ |  |
| $n^{FBP}$ | 1.80×10^0^ |  |
| $v_{max}$ | 4.62×10^-2^ | mM min^-1^ |
| $k_{pep}^{dp}$ | 1.10×10^0^ | mM |
| $k_{min}^{dp}$ | 8.00×10^-2^ | mM |
| $\alpha^{dp}$ | 1.00×10^0^ |  |
| $\alpha^{p}$ | 1.10×10^0^ |  |
| $\alpha_{end}$ | 1.00×10^0^ |  |
| $k_{fbp}^{dp}$ | 1.60×10^-4^ | mM |
| $k_{fbp}^{p}$ | 3.50×10^-4^ | mM |
| $\alpha_{base}^{dp}$ | 8.00×10^-2^ |  |
| $\alpha_{base}^{p}$ | 4.00×10^-2^ |  |
| $k_{ADP}$ | 2.30×10^0^ | mM |

Phosphofructokinase is one of several integral enzymes involved in hepatic glycolysis as it has an interconvertible phosphorylation state denoted$\gamma$. If $\gamma$ = 1, the enzyme is modelled in its phosphorylated state and therefore simulates the presence of the hormone, insulin, leading to increased glycolytic flux. If $\gamma$ = 0, the enzyme is modelled in its dephosphorylated state and therefore simulates the presence of the hormones epinephrine and glucagon which reduce glycolytic flux. When modelling carcinoma cells such as HepG2 cells, glycolytic flux is increased compared to that of normal cells due to the Warburg effect explained earlier. Therefore, when modelling hepatocarcinoma glycolytic flux, any interconvertible phosphorylation states kinetic equations use $\gamma$ = 1 (33).

**Lactate dehydrogenase (LDH):**


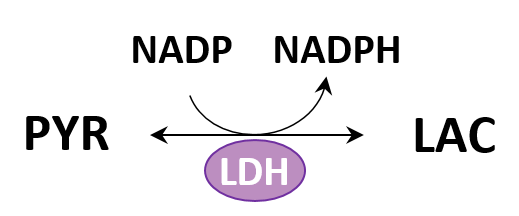


| $J_{LDH} =\frac{\frac{v_{max}}{k_{m}^{PYR} k_{m}^{NADH}}\left( \left[ PYR \right]\left[ NADH \right]-\frac{\left[ LAC \right][NAD]}{K_{eq}} \right)}{\left( 1+\frac{\left[ NADH \right]}{k_{m}^{NADH}} \right)\begin{aligned} \left( 1+\frac{\left[ PYR \right]}{k_{m}^{PYR}} \right) \end{aligned}\begin{aligned} \left( 1+\frac{\left[ LAC \right]}{k_{m}^{LAC}} \right) \end{aligned}\begin{aligned} \left( 1+\frac{\left[ NAD \right]}{k_{m}^{NAD}} \right)-1 \end{aligned}} .$ |
| --- |

| **Parameter** | **Value** | **Units** |
| --- | --- | --- |
| $K_{eq}$ | 2.78×10^-4^ |  |
| $k_{m}^{PYR}$ | 4.95×10^-1^ | mM |
| $v_{max}$ | 1.26×10^-2^ | mM min^-1^ |
| $k_{m}^{NADH}$ | 2.70×10^-2^ | mM |
| $k_{m}^{LAC}$ | 3.20×10^-1^ | mM |
| $k_{m}^{NAD}$ | 9.84×10^-1^ | mM |

**Glucose transporter 2 (GLUT2):**


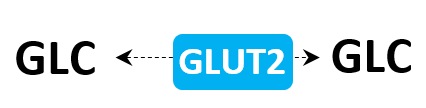

$$J_{GLUT2} =\frac{\frac{v_{max}}{k_{m}^{GLC}}\left( \left[ GLC_{e} \right]-\frac{\left[ GLC \right]}{K_{eq}} \right)}{\begin{aligned} \begin{aligned} 1+\frac{\left[ GLC_{e} \right]}{k_{m}^{GLC}} \end{aligned}+ \end{aligned}\frac{\left[ GLC \right]}{k_{m}^{GLC}}} .$$

| **Parameter** | **Value** | **Units** |
| --- | --- | --- |
| $K_{eq}$ | 1.00×10^0^ |  |
| $k_{m}^{GLC}$ | 4.20×10^1^ | mM |
| $v_{max}$ | 4.20×10^-3^ | mM min^-1^ |

**Monocarboxylate transporter 1 (LACT):**


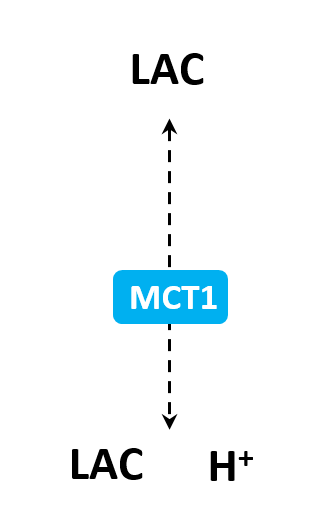

$$J_{LACT} =\frac{\frac{v_{max}}{k_{m}^{LAC}}\left( \left[ LAC_{e} \right]-\frac{\left[ LAC \right]}{K_{eq}} \right)}{\begin{aligned} \begin{aligned} 1+\frac{\left[ LAC_{e} \right]}{k_{m}^{LAC}} \end{aligned}+ \end{aligned}\frac{\left[ LAC \right]}{k_{m}^{LAC}}} .$$

| **Parameter** | **Value** | **Units** |
| --- | --- | --- |
| $K_{eq}$ | 1.00×10^0^ |  |
| $k_{m}^{LAC}$ | 8.00×10^-1^ | mM |
| $v_{max}$ | 5.42×10^-3^ | mM min^-1^ |

Monocarboxylate transporter 1 is the liver specific enzyme responsible for transport of lactic acid (40). Lactate is transported in its anionic form as LAC^-^ with co-transportation of a proton. This is due to the lactic acid having a pKa value of 3.82 which as a result means at pH 7.4 this weak carboxylic acid will exist in its dissociated form. This is very important when modelling extracellular acidification.


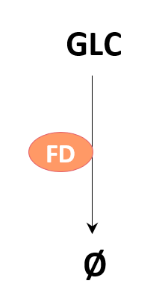
 **Glycogenolysis (FD):**

|  | \| **Parameter** \| **Value** \| **Units** \| \| --- \| --- \| --- \| \| $n$ \| 1.00×10^0^ \|  \| \| $k_{m}^{GLC}$ \| 9.00×10^0^ \| mM \| \| $v_{max}$ \| 1.00×10^0^ \| mM min^-1^ \|   $J_{FD} =\frac{v_{max} \left[ GLC \right]^{n}}{{k_{m}^{GLC}}^{n}+\left[ GLC \right]^{n}} .$ |
| --- | --- | --- | --- | --- | --- | --- | --- | --- | --- | --- | --- | --- | --- |

Glycogen storage in HepG2 cells is almost non-existent due to effects previously described in this chapter. However, there will be a level of storage and glucose regulation within the cell that exists outside the scope of this model. To account for intracellular glucose regulation, the essence of glycogenolysis has been captured using the glycogenolysis term *J_FD_*. This expression is used to prevent intracellular glucose concentrations from exceeding physiological levels of intracellular glucose for the model. HepG2 cells in standard *in vitro* cell culture are exposed to glucose concentrations approximately 5-fold higher than physiologically realistic levels (32,33). In order to prevent intracellular glucose concentration from inevitably equilibrating to that of the extracellular environment, a *k_m_* value of 9 mM has been estimated (hyperglycaemic condition). The other two parameters *n* and *v_max_* have been assigned arbitrary values of 10 and 1 respectively for the initial model, with the view to amend these values to match experimental data in later work.

**Appendix C: Model thermodynamics & pH calculations**

The model was constructed using the Biochemical Simulation Environment (BISEN), an open source computational toolset used to generate large ODE models in Matlab, capable of accounting for biochemical thermodynamics, rapid equilibria of multiple species while generating a dynamic proton and metal ion profile (2). The software, user manual (tutorial examples) are available at [www.bbc.mcw.edu/BISEN](http://www.bbc.mcw.edu/BISEN). The interwoven thermodynamics and subsequent dynamic pH time course generation is in full within the publication “Dynamics of muscle glycogenolysis modelled with pH time course computation and pH-dependent reaction equilibria and enzyme kinetics” (29).

Briefly, the state variables refer to the biochemical reactants within the model which are the sum of its interconvertible biochemical species. For example, ATP is a *reactant* that represents the sum of its related *species*: ATP^4-^, HATP^3-^, MgATP^2-^ etc. By accounting for the rapid interconversion of all species with metal ions and protons, we are able to model the differences in state depending upon the pH, as well as accounting for a complete proton stoichiometry. Each biochemical equation has its own associated equilibrium constant and Gibbs standard-state free energy of reaction ∆_r_G^0^ that is independent of pH yet dependent upon changes to temperature and ionic strength. Overall, this allows favorability of a reaction to change as a result of a pH change (28).

**Appendix D: *in vitro* data statistical analysis**

Significant changes between extracellular glucose concentrations were determined using Prism software as described in the methods section. One-way analysis of variance was determined using a one-way ANOVA with a Newman-Keuls Multiple comparison test to determine significance between all concentrations of glucose.

***ECAR analysis***

| \| Table Analyzed \| ECAR Normalized \| \|  \|  \| \| --- \| --- \| --- \| --- \| --- \| \|  \|  \|  \|  \|  \| \| One-way analysis of variance \|  \|  \|  \|  \| \| P value \| P<0.0001 \|  \|  \|  \| \| P value summary \| *** \|  \|  \|  \| \| Are means signif. different? (P < 0.05) \| Yes \|  \|  \|  \| \| Number of groups \| 10 \|  \|  \|  \| \| F \| 384.2 \|  \|  \|  \| \| R squared \| 0.9665 \|  \|  \|  \| \|  \|  \|  \|  \|  \| \| Bartlett's test for equal variances \|  \|  \|  \|  \| \| Bartlett's statistic (corrected) \| 5.51 \|  \|  \|  \| \| P value \| 0.7877 \|  \|  \|  \| \| P value summary \| ns \|  \|  \|  \| \| Do the variances differ signif. (P < 0.05) \| No \|  \|  \|  \| \|  \|  \|  \|  \|  \| \| ANOVA Table \| SS \| df \| MS \|  \| \| Treatment (between columns) \| 2.287 \| 9 \| 0.2541 \|  \| \| Residual (within columns) \| 0.07937 \| 120 \| 0.000661 \|  \| \| Total \| 2.366 \| 129 \|  \|  \| \|  \|  \|  \|  \|  \| \| Newman-Keuls Multiple Comparison Test \| Mean Diff. \| q \| Significant? P < 0.001? \| Summary \| \| Concentration (mM) \|  \|  \|  \|  \| \| 0 vs 5.0 \| -0.5022 \| 70.4 \| Yes \| *** \| \| 0 vs 12.5 \| -0.3569 \| 50.04 \| Yes \| *** \| \| 0 vs 15.0 \| -0.3478 \| 48.76 \| Yes \| *** \| \| 0 vs 25.0 \| -0.3404 \| 47.72 \| Yes \| *** \| \| 0 vs 10.0 \| -0.3137 \| 43.98 \| Yes \| *** \| \| 0 vs 2.5 \| -0.2827 \| 39.63 \| Yes \| *** \| \| 0 vs 7.5 \| -0.2442 \| 34.23 \| Yes \| *** \| \| 0 vs 1 \| -0.2095 \| 29.38 \| Yes \| *** \| \| 0 vs 0.1 \| -0.1145 \| 16.05 \| Yes \| *** \| \| 0.1 vs 5.0 \| -0.3877 \| 54.35 \| Yes \| *** \| \| 0.1 vs 12.5 \| -0.2425 \| 33.99 \| Yes \| *** \| \| 0.1 vs 15.0 \| -0.2333 \| 32.71 \| Yes \| *** \| \| 0.1 vs 25.0 \| -0.2259 \| 31.67 \| Yes \| *** \| \| 0.1 vs 10.0 \| -0.1992 \| 27.93 \| Yes \| *** \| \| 0.1 vs 2.5 \| -0.1682 \| 23.59 \| Yes \| *** \| \| 0.1 vs 7.5 \| -0.1297 \| 18.18 \| Yes \| *** \| \| 0.1 vs 1 \| -0.09508 \| 13.33 \| Yes \| *** \| \| 1 vs 5.0 \| -0.2926 \| 41.02 \| Yes \| *** \| \| 1 vs 12.5 \| -0.1474 \| 20.66 \| Yes \| *** \| \| 1 vs 15.0 \| -0.1382 \| 19.38 \| Yes \| *** \| \| 1 vs 25.0 \| -0.1308 \| 18.34 \| Yes \| *** \| \| 1 vs 10.0 \| -0.1042 \| 14.6 \| Yes \| *** \| \| 1 vs 2.5 \| -0.07315 \| 10.26 \| Yes \| *** \| \| 1 vs 7.5 \| -0.03462 \| 4.853 \| Yes \| *** \| \| 7.5 vs 5.0 \| -0.258 \| 36.17 \| Yes \| *** \| \| 7.5 vs 12.5 \| -0.1128 \| 15.81 \| Yes \| *** \| \| 7.5 vs 15.0 \| -0.1036 \| 14.53 \| Yes \| *** \| \| 7.5 vs 25.0 \| -0.09623 \| 13.49 \| Yes \| *** \| \| 7.5 vs 10.0 \| -0.06954 \| 9.749 \| Yes \| *** \| \| 7.5 vs 2.5 \| -0.03854 \| 5.403 \| Yes \| *** \| \| 2.5 vs 5.0 \| -0.2195 \| 30.77 \| Yes \| *** \| \| 2.5 vs 12.5 \| -0.07423 \| 10.41 \| Yes \| *** \| \| 2.5 vs 15.0 \| -0.06508 \| 9.124 \| Yes \| *** \| \| 2.5 vs 25.0 \| -0.05769 \| 8.088 \| Yes \| *** \| \| 2.5 vs 10.0 \| -0.031 \| 4.346 \| No \| ** \| \| 10.0 vs 5.0 \| -0.1885 \| 26.42 \| Yes \| *** \| \| 10.0 vs 12.5 \| -0.04323 \| 6.061 \| Yes \| *** \| \| 10.0 vs 15.0 \| -0.03408 \| 4.777 \| No \| ** \| \| 10.0 vs 25.0 \| -0.02669 \| 3.742 \| No \| ** \| \| 25.0 vs 5.0 \| -0.1618 \| 22.68 \| Yes \| *** \| \| 25.0 vs 12.5 \| -0.01654 \| 2.319 \| No \| ns \| \| 25.0 vs 15.0 \| -0.00739 \| --- \| No \| ns \| \| 15.0 vs 5.0 \| -0.1544 \| 21.64 \| Yes \| *** \| \| 15.0 vs 12.5 \| -0.00915 \| --- \| No \| ns \| \| 12.5 vs 5.0 \| -0.1452 \| 20.36 \| Yes \| *** \| |
| --- | --- | --- | --- | --- | --- | --- | --- | --- | --- | --- | --- | --- | --- | --- | --- | --- | --- | --- | --- | --- | --- | --- | --- | --- | --- | --- | --- | --- | --- | --- | --- | --- | --- | --- | --- | --- | --- | --- | --- | --- | --- | --- | --- | --- | --- | --- | --- | --- | --- | --- | --- | --- | --- | --- | --- | --- | --- | --- | --- | --- | --- | --- | --- | --- | --- | --- | --- | --- | --- | --- | --- | --- | --- | --- | --- | --- | --- | --- | --- | --- | --- | --- | --- | --- | --- | --- | --- | --- | --- | --- | --- | --- | --- | --- | --- | --- | --- | --- | --- | --- | --- | --- | --- | --- | --- | --- | --- | --- | --- | --- | --- | --- | --- | --- | --- | --- | --- | --- | --- | --- | --- | --- | --- | --- | --- | --- | --- | --- | --- | --- | --- | --- | --- | --- | --- | --- | --- | --- | --- | --- | --- | --- | --- | --- | --- | --- | --- | --- | --- | --- | --- | --- | --- | --- | --- | --- | --- | --- | --- | --- | --- | --- | --- | --- | --- | --- | --- | --- | --- | --- | --- | --- | --- | --- | --- | --- | --- | --- | --- | --- | --- | --- | --- | --- | --- | --- | --- | --- | --- | --- | --- | --- | --- | --- | --- | --- | --- | --- | --- | --- | --- | --- | --- | --- | --- | --- | --- | --- | --- | --- | --- | --- | --- | --- | --- | --- | --- | --- | --- | --- | --- | --- | --- | --- | --- | --- | --- | --- | --- | --- | --- | --- | --- | --- | --- | --- | --- | --- | --- | --- | --- | --- | --- | --- | --- | --- | --- | --- | --- | --- | --- | --- | --- | --- | --- | --- | --- | --- | --- | --- | --- | --- | --- | --- | --- | --- | --- | --- | --- | --- | --- | --- | --- | --- | --- | --- | --- | --- | --- | --- | --- | --- | --- | --- | --- | --- | --- | --- | --- | --- | --- | --- | --- | --- | --- | --- | --- | --- | --- | --- | --- | --- | --- | --- | --- | --- | --- | --- | --- | --- | --- | --- | --- | --- | --- | --- | --- | --- | --- | --- | --- | --- | --- | --- | --- | --- | --- | --- | --- | --- | --- | --- | --- | --- | --- | --- | --- | --- | --- | --- |
| **Table S5:** Statistical analysis of *in vitro* EFA using different concentrations of glucose. Significance between average ECAR normalised to basal ECAR and protein content are shown, with *P*-values < 0.05 = *, *P*-values < 0.01 = ** and *P*-values < 0.001 = ***. |

All concentrations of extracellular glucose were significant when compared to the control where the extracellular concentration of glucose is equal to zero (Figure S4).

|  *********  *********  *********  *********  *********  *********  *********  *********  ********* |
| --- |
| **Figure S2:** **Statistical analysis of *in vitro* EFA ECAR**. Anova with Newman-Keuls Multiple comparison test for different concentrations of glucose showing all concentrations (mean ± SD) used above zero were significant compared to 0 mM control. *P*-values < 0.001 = ***. |

***OCR analysis***

| \| Table Analyzed \| OCR Normalized \| \|  \|  \|  \| \| --- \| --- \| --- \| --- \| --- \| --- \| \|  \|  \|  \|  \|  \|  \| \| One-way analysis of variance \|  \|  \|  \|  \|  \| \| P value \| P<0.0001 \|  \|  \|  \|  \| \| P value summary \| *** \|  \|  \|  \|  \| \| Are means signif. different? (P < 0.05) \| Yes \|  \|  \|  \|  \| \| Number of groups \| 10 \|  \|  \|  \|  \| \| F \| 15.88 \|  \|  \|  \|  \| \| R squared \| 0.5436 \|  \|  \|  \|  \| \|  \|  \|  \|  \|  \|  \| \| Bartlett's test for equal variances \|  \|  \|  \|  \|  \| \| Bartlett's statistic (corrected) \| 6.709 \|  \|  \|  \|  \| \| P value \| 0.6674 \|  \|  \|  \|  \| \| P value summary \| ns \|  \|  \|  \|  \| \| Do the variances differ signif. (P < 0.05) \| No \|  \|  \|  \|  \| \|  \|  \|  \|  \|  \|  \| \| ANOVA Table \| SS \| df \| MS \|  \|  \| \| Treatment (between columns) \| 0.1026 \| 9 \| 0.0114 \|  \|  \| \| Residual (within columns) \| 0.08615 \| 120 \| 0.000718 \|  \|  \| \| Total \| 0.1887 \| 129 \|  \|  \|  \| \|  \|  \|  \|  \|  \|  \| \| Newman-Keuls Multiple Comparison Test \| Mean Diff. \| q \| Significant? P < 0.001? \| Summary \|  \| \| Concentration (mM) \|  \|  \|  \|  \|  \| \| 25.0 vs 0 \| -0.08932 \| 12.02 \| Yes \| *** \|  \| \| 25.0 vs 0.1 \| -0.06621 \| 8.909 \| Yes \| *** \|  \| \| 25.0 vs 1 \| -0.06442 \| 8.669 \| Yes \| *** \|  \| \| 25.0 vs 2.5 \| -0.04373 \| 5.885 \| No \| ** \|  \| \| 25.0 vs 5.0 \| -0.02905 \| 3.909 \| No \| ns \|  \| \| 25.0 vs 12.5 \| -0.02263 \| --- \| No \| ns \|  \| \| 25.0 vs 15.0 \| -0.01409 \| --- \| No \| ns \|  \| \| 25.0 vs 10.0 \| -0.01156 \| --- \| No \| ns \|  \| \| 25.0 vs 7.5 \| -0.01 \| --- \| No \| ns \|  \| \| 7.5 vs 0 \| -0.07932 \| 10.67 \| Yes \| *** \|  \| \| 7.5 vs 0.1 \| -0.05621 \| 7.563 \| Yes \| *** \|  \| \| 7.5 vs 1 \| -0.05442 \| 7.323 \| Yes \| *** \|  \| \| 7.5 vs 2.5 \| -0.03373 \| 4.539 \| No \| * \|  \| \| 7.5 vs 5.0 \| -0.01905 \| --- \| No \| ns \|  \| \| 7.5 vs 12.5 \| -0.01263 \| --- \| No \| ns \|  \| \| 7.5 vs 15.0 \| -0.00409 \| --- \| No \| ns \|  \| \| 7.5 vs 10.0 \| -0.00156 \| --- \| No \| ns \|  \| \| 10.0 vs 0 \| -0.07776 \| 10.46 \| Yes \| *** \|  \| \| 10.0 vs 0.1 \| -0.05464 \| 7.353 \| Yes \| *** \|  \| \| 10.0 vs 1 \| -0.05286 \| 7.113 \| Yes \| *** \|  \| \| 10.0 vs 2.5 \| -0.03217 \| 4.329 \| No \| * \|  \| \| 10.0 vs 5.0 \| -0.01749 \| --- \| No \| ns \|  \| \| 10.0 vs 12.5 \| -0.01106 \| --- \| No \| ns \|  \| \| 10.0 vs 15.0 \| -0.00253 \| --- \| No \| ns \|  \| \| 15.0 vs 0 \| -0.07523 \| 10.12 \| Yes \| *** \|  \| \| 15.0 vs 0.1 \| -0.05212 \| 7.014 \| Yes \| *** \|  \| \| 15.0 vs 1 \| -0.05033 \| 6.773 \| Yes \| *** \|  \| \| 15.0 vs 2.5 \| -0.02964 \| 3.989 \| No \| * \|  \| \| 15.0 vs 5.0 \| -0.01496 \| --- \| No \| ns \|  \| \| 15.0 vs 12.5 \| -0.00854 \| --- \| No \| ns \|  \| \| 12.5 vs 0 \| -0.06669 \| 8.975 \| Yes \| *** \|  \| \| 12.5 vs 0.1 \| -0.04358 \| 5.864 \| Yes \| *** \|  \| \| 12.5 vs 1 \| -0.04179 \| 5.624 \| Yes \| *** \|  \| \| 12.5 vs 2.5 \| -0.0211 \| 2.84 \| No \| ns \|  \| \| 12.5 vs 5.0 \| -0.00642 \| --- \| No \| ns \|  \| \| 5.0 vs 0 \| -0.06027 \| 8.111 \| Yes \| *** \|  \| \| 5.0 vs 0.1 \| -0.03716 \| 5 \| No \| ** \|  \| \| 5.0 vs 1 \| -0.03537 \| 4.76 \| No \| ** \|  \| \| 5.0 vs 2.5 \| -0.01468 \| --- \| No \| ns \|  \| \| 2.5 vs 0 \| -0.04559 \| 6.135 \| Yes \| *** \|  \| \| 2.5 vs 0.1 \| -0.02248 \| 3.025 \| No \| ns \|  \| \| 2.5 vs 1 \| -0.02069 \| --- \| No \| ns \|  \| \| 1 vs 0 \| -0.0249 \| 3.351 \| No \| ns \|  \| \| 1 vs 0.1 \| -0.00179 \| --- \| No \| ns \|  \| \| 0.1 vs 0 \| -0.02311 \| --- \| No \| ns \|  \| |
| --- | --- | --- | --- | --- | --- | --- | --- | --- | --- | --- | --- | --- | --- | --- | --- | --- | --- | --- | --- | --- | --- | --- | --- | --- | --- | --- | --- | --- | --- | --- | --- | --- | --- | --- | --- | --- | --- | --- | --- | --- | --- | --- | --- | --- | --- | --- | --- | --- | --- | --- | --- | --- | --- | --- | --- | --- | --- | --- | --- | --- | --- | --- | --- | --- | --- | --- | --- | --- | --- | --- | --- | --- | --- | --- | --- | --- | --- | --- | --- | --- | --- | --- | --- | --- | --- | --- | --- | --- | --- | --- | --- | --- | --- | --- | --- | --- | --- | --- | --- | --- | --- | --- | --- | --- | --- | --- | --- | --- | --- | --- | --- | --- | --- | --- | --- | --- | --- | --- | --- | --- | --- | --- | --- | --- | --- | --- | --- | --- | --- | --- | --- | --- | --- | --- | --- | --- | --- | --- | --- | --- | --- | --- | --- | --- | --- | --- | --- | --- | --- | --- | --- | --- | --- | --- | --- | --- | --- | --- | --- | --- | --- | --- | --- | --- | --- | --- | --- | --- | --- | --- | --- | --- | --- | --- | --- | --- | --- | --- | --- | --- | --- | --- | --- | --- | --- | --- | --- | --- | --- | --- | --- | --- | --- | --- | --- | --- | --- | --- | --- | --- | --- | --- | --- | --- | --- | --- | --- | --- | --- | --- | --- | --- | --- | --- | --- | --- | --- | --- | --- | --- | --- | --- | --- | --- | --- | --- | --- | --- | --- | --- | --- | --- | --- | --- | --- | --- | --- | --- | --- | --- | --- | --- | --- | --- | --- | --- | --- | --- | --- | --- | --- | --- | --- | --- | --- | --- | --- | --- | --- | --- | --- | --- | --- | --- | --- | --- | --- | --- | --- | --- | --- | --- | --- | --- | --- | --- | --- | --- | --- | --- | --- | --- | --- | --- | --- | --- | --- | --- | --- | --- | --- | --- | --- | --- | --- | --- | --- | --- | --- | --- | --- | --- | --- | --- | --- | --- | --- | --- | --- | --- | --- | --- | --- | --- | --- | --- | --- | --- | --- | --- | --- | --- | --- | --- | --- | --- | --- | --- | --- | --- | --- | --- | --- | --- | --- | --- | --- | --- | --- | --- | --- | --- | --- | --- | --- | --- | --- | --- | --- | --- | --- | --- | --- | --- | --- | --- | --- | --- | --- | --- | --- | --- | --- | --- | --- | --- | --- | --- | --- | --- | --- | --- | --- | --- | --- | --- | --- | --- | --- | --- | --- | --- | --- | --- | --- | --- | --- | --- | --- | --- | --- | --- | --- | --- | --- | --- | --- | --- | --- | --- | --- | --- | --- | --- | --- | --- | --- | --- |
| **Table S6:** Statistical analysis of *in vitro* EFA using different concentrations of glucose. Significance between average OCR normalised to basal OCR and protein content are shown, with *P*-values < 0.05 = *, *P*-values < 0.01 = ** and *P*-values < 0.001 = ***. |

Significance between 0 mM and all other concentrations of extracellular glucose is shown in Figure S3.

|  *********  *********  *********  *********  *********  *********  *********  ********* |
| --- |
| **Figure S3** **Statistical analysis of *in vitro* EFA OCR**. Anova with Newman-Keuls Multiple comparison test for different concentrations of glucose showing all concentrations (mean ± SD) used above zero were significant compared to 0 mM control. *P*-values < 0.001 = ***. |

**Appendix E**

***Changes in NAD^+^/NADH redox state perturb glycolytic rate and energy production.***

The sensitivity of glycolytic rate and energy production to changes in the free NAD^+^/NADH redox state were investigated by simulating NAD^+^/NADH ratios between 0.1 and 3500. This wide ratio range aims to capture the various ratios attributed to different cell types with different metabolic phenotypes [9,45].

Two different model outputs for glycolytic rate were expressed, PPR_gly_ and LDH flux, to determine whether pyruvate-to-lactate conversion was an adequate method of approximating glycolytic rate, compared to a direct simulation of extracellular acidification. The model was simulated to steady state (not shown), assigned a new NAD^+^/NADH ratio at t = 0, then subsequently simulated for 120 min with NAD^+^/NAD fixed at this ratio. Figure 8A shows the percentage variations in glycolytic rate compared to no changes in redox state (black dashed line) for these different NAD^+^/NADH ratios, with simulations suggesting a dependent relationship between redox state and PPR_gly_. Increases in NAD^+^/NADH yield a maximum increase in glycolytic rate of 6.1%, while decreases in the NAD^+^/NADH ratio result in a maximum reduction of glycolytic rate of 8.9% over 120 min. Figure 8B reveals how the simulated ATP content behaves after perturbations in the NAD^+^/NADH ratio. These simulations suggest that ATP is more sensitive to variance in the redox ratio than glycolytic rate, i.e., there is a clear immediate change in ATP following the perturbation, whereas changes in PPR_gly_ manifest after 4.6 min (Figure 8A). Furthermore, after a 120-min simulation, the model shows that, while a maximum percentage change in ATP concentration of 17.8% is possible when the NAD^+^/NADH ratio tends towards 3500, ATP is much more sensitive to decreases in the redox state ratio whereby a 63.4% drop occurs when the NAD^+^/NADH ratio is 0.1. Figure 8C illustrates the LDH flux response during redox state ratio perturbations compared to no change (black dashed line). NAD^+^ and NADH are directly involved in the rate at which LDH converts pyruvate to lactate and as such, simulations show that perturbations in NAD^+^/NADH immediately alter the LDH flux, with effects ranging from -98.8% to 26.9% compared to no perturbation. The time-course profile for LDH is much more dynamic over 120 min compared to ATP and PPR_gly_ changes. Increases in the redox state ratio yield steady decreases in the LDH flux, whereas decreases in the NAD^+^/NADH ratio lead to an initial transient increase in LDH flux (up to 219.8%) followed by a decrease (where at t = 120, LDH flux is -25.5%). Changes in NAD^+^/NADH effect the conversion ratio of lactate and pyruvate such that, when the ratio is elevated, pyruvate formation from lactate is favoured. The opposite is true when the ratio is decreased, resulting in conversion of pyruvate to lactate instead. In Figure 8C, the transient increase in LDH flux at diminished NAD^+^/NADH ratios confirm that LDH is not at equilibrium, but does act to tend towards it, i.e. after a large increase in LDH flux of up to 219.8% compared to no perturbation, the ratio of LDH substrate pyruvate increases, resulting in a change in the reaction quotient Q leading to a decrease in LDH flux.


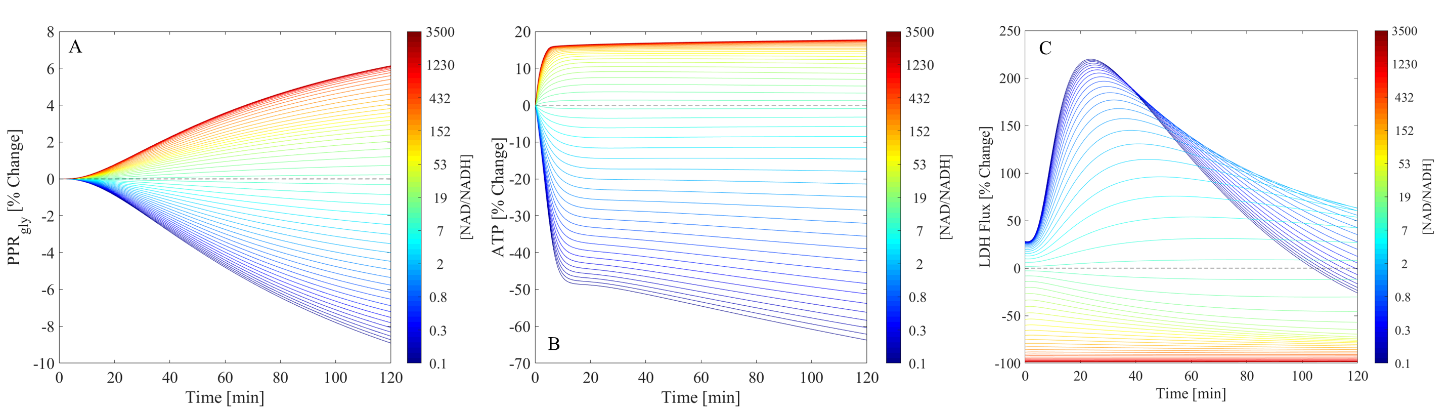


**Figure S4. NAD/NADH ratio perturbations.** Simulations of the resulting changes in glycolytic proton production rate (PPR_gly_), cytoplasmic ATP and Lactate dehydrogenase flux (LDH) as a function of perturbations in NAD^+^/NADH. A-C show the percentage change time-course profiles during a 120-min simulation as a function of variation in NAD^+^/NADH ratio compared to no changes (black dashed line). For all simulations, NAD^+^/NADH perturbation occurs at t = 0. Time *t* represents time post NAD^+^/NADH perturbation.


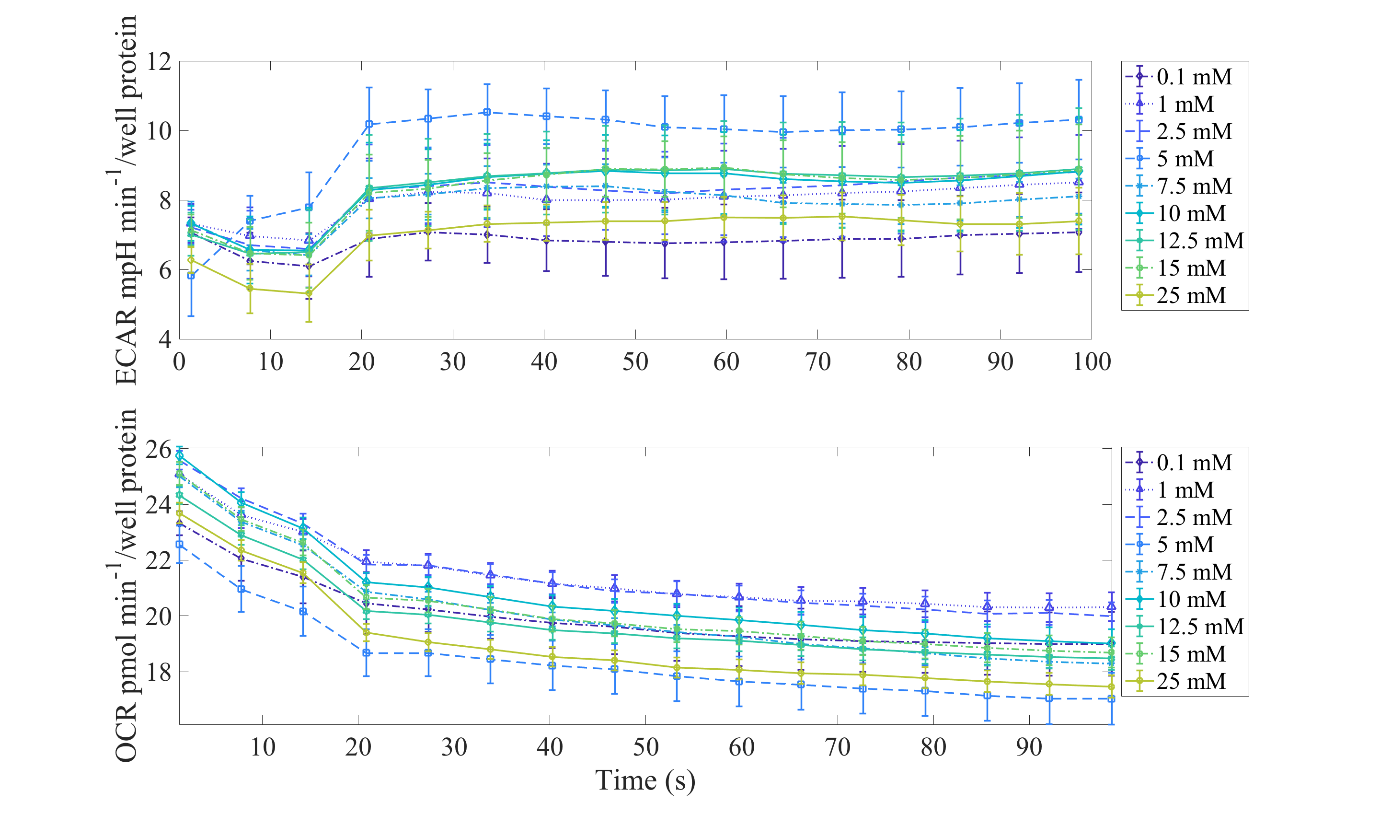


**Figure S5. *In vitro* EFA extracellular glucose results.** Changes in extracellular acidification rate (ECAR) and oxygen consumption rate (OCR), as a function of perturbations in extracellular glucose concentrations. HepG2 cells were exposed to 9 concentrations of glucose (0.1 – 25 mM) at t = 16 minutes, with the resulting OCR and ECAR data captured. Results show the variance of 4 experimental replicates (n = 4) expressed as average OCR and ECAR with their standard deviation. Data values are available in the EFA raw data.xlsx spreadsheet located in the S1 Data file.

**References**

1. König M, Bulik S, Holzhütter H-G. Quantifying the Contribution of the Liver to Glucose Homeostasis: A Detailed Kinetic Model of Human Hepatic Glucose Metabolism. PLoS computational biology. 2012;8(6):e1002577.

2. Vanlier J, Wu F, Qi F, Vinnakota KC, Han Y, Dash RK, et al. BISEN: Biochemical Simulation Environment. Bioinformatics. 2009;25(6):836-7.

3. S.A. Mookerjee, R.L.S. Goncalves, A.A. Gerencser, D.G. Nicholls, M.D. Brand, The contributions of respiration and glycolysis to extracellular acid production., Biochim. Biophys. Acta. 1847 (2015) 171–181. doi:10.1016/j.bbabio.2014.10.005.

4. Overmoyer B. Uniformity of liver density and nonheme (storate) iron distribution. Archives of pathology & laboratory medicine. 1987;111(6):5.

5. Gille C, Bölling C, Hoppe A, Bulik S, Hoffmann S, Hübner K, et al. HepatoNet1: a comprehensive metabolic reconstruction of the human hepatocyte for the analysis of liver physiology. Molecular Systems Biology. 2010;6(1).

6. Vinnakota K, Kemp ML, Kushmerick MJ. Dynamics of muscle glycogenolysis modeled with pH time course computation and pH-dependent reaction equilibria and enzyme kinetics. Biophysical journal. 2006;91(4):1264-87.

7. Daniel. A. Beard HQ. Chemical Biophysics. W. Mark Saltzman SC, editor. Cambridge Texts in Biomedical Engineering: Chambridge Texts; 2008. 311 p.
