# Supplementary material for: Modelling the impact of changes in the extracellular environment on the cytosolic free NAD+/NADH ratio during cell culture
Source: PLoS One. 2018 Nov 29;13(11):e0207803. doi: 10.1371/journal.pone.0207803 (PMC6264472; doi:10.1371/journal.pone.0207803)
Supplement: S2 File — Zip file containing html versions of model code. (ZIP) [file pone.0207803.s004.zip › html/dXdT_HGM.html]

dXdT\_HGM 

## Contents

- HEPATOCARCINOMA GLYCOLYSIS MODEL
- GLOBAL VARIABLES
- LIST OF STATE VARIABLES
- THERMODYNAMIC DATA
- STATE VARIABLES
- DISSOCIATION CONSTANTS
- BINDING POLYNOMIALS
- THERMODYNAMIC EQUATIONS
- FLUX EQUATIONS
- GLK\_cytoplasm
- G6PASE\_cytoplasm
- PGI\_cytoplasm
- PFK\_cytoplasm
- FBP1\_cytoplasm
- ALD\_cytoplasm
- TPI\_cytoplasm
- GAPDH\_cytoplasm
- PGK\_cytoplasm
- PGYM\_cytoplasm
- ENO\_cytoplasm
- PYK\_cytoplasm
- LDH\_cytoplasm
- FD\_cytoplasm
- GLUT2:extracellular\_to\_cytoplasm
- LACT:cytoplasm\_to\_extracellular
- REACTANT TIME DERIVATIVES
- ION EQUATIONS
- ELECTROPHYS EQUATIONS
- FLUX VECTOR:

## HEPATOCARCINOMA GLYCOLYSIS MODEL

```
author: Ross Kelly
        Department of Applied Mathematics
        Liverpool John Moores University
        R.A.Kelly@ljmu.ac.uk
date:   23.08.2018
```

```
% Output parameters:
%   f     time derivatives of the model
%   J     flux

% Mandatory input parameters:
%   t     time
%   x     state variables at t=0
%   T     temperature in degreesCelcius
%   BX    buffer sizes
%   K_BX  proton buffer dissociation constants ( cytoplasm extracellular )
%   par   parameter vector for the free parameters


function [f,J] = dXdT_HGM(t,x,T,BX,K_BX,~,kfbp1)
```

## GLOBAL VARIABLES

temperature 37

```
% Set proton buffer sizes
BX(1) = 2.0e+4; % uM
BX(2) = 2.0e+4; % uM

% Set proton buffer binding constants
K_BX(1) = 1e-7; % uM
K_BX(2) = 1e-7; % uM
```

## LIST OF STATE VARIABLES

1 glucose\_cytoplasm 2 ATP\_cytoplasm 3 ADP\_cytoplasm 4 glucose6phos\_cytoplasm 5 fructose6phos\_cytoplasm 6 Pi\_cytoplasm 7 fructose16phos\_cytoplasm 8 fructose26phos\_cytoplasm 9 dihydroxyacetonephos\_cytoplasm 10 glyceraldehydephos\_cytoplasm 11 NAD\_cytoplasm 12 bpg\_cytoplasm 13 NADH\_cytoplasm 14 pg3\_cytoplasm 15 pg2\_cytoplasm 16 pep\_cytoplasm 17 pyruvate\_cytoplasm 18 lactate\_cytoplasm 19 glucose\_extracellular 20 lactate\_extracellular 21 h\_cytoplasm 22 m\_cytoplasm 23 k\_cytoplasm 24 h\_extracellular 25 m\_extracellular 26 k\_extracellular 27 DPsi\_extracellular\_to\_cytoplasm

```
% PARTIAL VOLUME FRACTIONS
VWater_cytoplasm = 1; % [=] l water (l region)^{-1}, Vinnakota and Bassingthwaighte, AJP, 2004
VRegion_cytoplasm = 0.0031; % [=] l region (l tissue)^{-1}, Vinnakota and Bassingthwaighte, AJP, 2004
VWater_extracellular = 1; % [=] l water (l region)^{-1}, Vinnakota and Bassingthwaighte, AJP, 2004
VRegion_extracellular = 0.9969; % [=] l region (l tissue)^{-1}, Vinnakota and Bassingthwaighte, AJP, 2004
```

## THERMODYNAMIC DATA

```
RT = 8.314*(T+273.15)/1e3; % kJ  mol^{-1}
F = 0.096484; % kJ mol^{-1} mV^{-1}
```

## STATE VARIABLES

```
% Concentrations of Reference Species
glucose_cytoplasm = x(1);
ATP_cytoplasm = x(2);
ADP_cytoplasm = x(3);
glucose6phos_cytoplasm = x(4);
fructose6phos_cytoplasm = x(5);
Pi_cytoplasm = x(6);
fructose16phos_cytoplasm = x(7);
fructose26phos_cytoplasm = x(8);
dihydroxyacetonephos_cytoplasm = x(9);
glyceraldehydephos_cytoplasm = x(10);
NAD_cytoplasm = x(11);
bpg_cytoplasm = x(12);
NADH_cytoplasm = x(13);
pg3_cytoplasm = x(14);
pg2_cytoplasm = x(15);
pep_cytoplasm = x(16);
pyruvate_cytoplasm = x(17);
lactate_cytoplasm = x(18);
glucose_extracellular = x(19);
lactate_extracellular = x(20);
% Concentrations of H, Mg, and K
h_cytoplasm = x(21);
m_cytoplasm = x(22);
k_cytoplasm = x(23);
h_extracellular = x(24);
m_extracellular = x(25);
k_extracellular = x(26);

% Membrane potentials
DPsi_extracellular_to_cytoplasm = x(27);
```

## DISSOCIATION CONSTANTS

glucose\_cytoplasm

```
Kh(1) = Inf;
Km(1) = Inf;
Kk(1) = Inf;
% ATP_cytoplasm
Kh(2) = 2.7566016511811682e-07;
Km(2) = 8.4303632255990519e-05;
Kk(2) = 0.09708512798373839;
% ADP_cytoplasm
Kh(3) = 4.1057373286278706e-07;
Km(3) = 0.00071485288102034542;
Kk(3) = 0.1319048728526625;
% glucose6phos_cytoplasm
Kh(4) = 1.5423268198693262e-06;
Km(4) = 0.022805651456439764;
Kk(4) = Inf;
% fructose6phos_cytoplasm
Kh(5) = 1.5423268198693262e-06;
Km(5) = 0.022805651456439764;
Kk(5) = Inf;
% Pi_cytoplasm
Kh(6) = 2.1535140068037538e-07;
Km(6) = 0.018897823092027442;
Kk(6) = 0.38034165601214515;
% fructose16phos_cytoplasm
Kh(7) = 3.3139639988698244e-07;
Km(7) = 0.0041753758246859621;
Kk(7) = Inf;
% fructose26phos_cytoplasm
Kh(8) = Inf;
Km(8) = Inf;
Kk(8) = Inf;
% dihydroxyacetonephos_cytoplasm
Kh(9) = 1.5141674051862649e-06;
Km(9) = 0.038935681994297811;
Kk(9) = Inf;
% glyceraldehydephos_cytoplasm
Kh(10) = 6.4591280308941423e-06;
Km(10) = Inf;
Kk(10) = Inf;
% NAD_cytoplasm
Kh(11) = Inf;
Km(11) = Inf;
Kk(11) = Inf;
% bpg_cytoplasm
Kh(12) = 4.5745437575003546e-08;
Km(12) = Inf;
Kk(12) = Inf;
% NADH_cytoplasm
Kh(13) = Inf;
Km(13) = Inf;
Kk(13) = Inf;
% pg3_cytoplasm
Kh(14) = 1.6992639331858257e-07;
Km(14) = 0.010728071992282361;
Kk(14) = 0.17793477751121317;
% pg2_cytoplasm
Kh(15) = 1.3190487285266262e-07;
Km(15) = 0.0061733610749874698;
Kk(15) = 0.087148685254029176;
% pep_cytoplasm
Kh(16) = 7.5034473514821362e-07;
Km(16) = 0.009561404227488024;
Kk(16) = 0.10971369447074888;
% pyruvate_cytoplasm
Kh(17) = 0.0073747984080028035;
Km(17) = 0.09553750450747911;
Kk(17) = Inf;
% lactate_cytoplasm
Kh(18) = 0.00023602950579441515;
Km(18) = 0.12594295910184752;
Kk(18) = Inf;
% glucose_extracellular
Kh(19) = Inf;
Km(19) = Inf;
Kk(19) = Inf;
% lactate_extracellular
Kh(20) = 0.00023602950579441515;
Km(20) = 0.12594295910184752;
Kk(20) = Inf;
```

## BINDING POLYNOMIALS

```
P( 1 ) = 1  + h_cytoplasm/Kh(1) + m_cytoplasm/Km(1) + k_cytoplasm/Kk(1);
P( 2 ) = 1  + h_cytoplasm/Kh(2) + m_cytoplasm/Km(2) + k_cytoplasm/Kk(2);
P( 3 ) = 1  + h_cytoplasm/Kh(3) + m_cytoplasm/Km(3) + k_cytoplasm/Kk(3);
P( 4 ) = 1  + h_cytoplasm/Kh(4) + m_cytoplasm/Km(4) + k_cytoplasm/Kk(4);
P( 5 ) = 1  + h_cytoplasm/Kh(5) + m_cytoplasm/Km(5) + k_cytoplasm/Kk(5);
P( 6 ) = 1  + h_cytoplasm/Kh(6) + m_cytoplasm/Km(6) + k_cytoplasm/Kk(6);
P( 7 ) = 1  + h_cytoplasm/Kh(7) + m_cytoplasm/Km(7) + k_cytoplasm/Kk(7);
P( 8 ) = 1  + h_cytoplasm/Kh(8) + m_cytoplasm/Km(8) + k_cytoplasm/Kk(8);
P( 9 ) = 1  + h_cytoplasm/Kh(9) + m_cytoplasm/Km(9) + k_cytoplasm/Kk(9);
P( 10 ) = 1  + h_cytoplasm/Kh(10) + m_cytoplasm/Km(10) + k_cytoplasm/Kk(10);
P( 11 ) = 1  + h_cytoplasm/Kh(11) + m_cytoplasm/Km(11) + k_cytoplasm/Kk(11);
P( 12 ) = 1  + h_cytoplasm/Kh(12) + m_cytoplasm/Km(12) + k_cytoplasm/Kk(12);
P( 13 ) = 1  + h_cytoplasm/Kh(13) + m_cytoplasm/Km(13) + k_cytoplasm/Kk(13);
P( 14 ) = 1  + h_cytoplasm/Kh(14) + m_cytoplasm/Km(14) + k_cytoplasm/Kk(14);
P( 15 ) = 1  + h_cytoplasm/Kh(15) + m_cytoplasm/Km(15) + k_cytoplasm/Kk(15);
P( 16 ) = 1  + h_cytoplasm/Kh(16) + m_cytoplasm/Km(16) + k_cytoplasm/Kk(16);
P( 17 ) = 1  + h_cytoplasm/Kh(17) + m_cytoplasm/Km(17) + k_cytoplasm/Kk(17);
P( 18 ) = 1  + h_cytoplasm/Kh(18) + m_cytoplasm/Km(18) + k_cytoplasm/Kk(18);
P( 19 ) = 1  + h_extracellular/Kh(19) + m_extracellular/Km(19) + k_extracellular/Kk(19);
P( 20 ) = 1  + h_extracellular/Kh(20) + m_extracellular/Km(20) + k_extracellular/Kk(20);
```

## THERMODYNAMIC EQUATIONS

```
DGro_GLK =17.5685;
DGro_G6PASE =-11.8607;
DGro_PGI =3.13;
DGro_PFK =NaN;
DGro_FBP1 =NaN;
DGro_ALD =24.944;
DGro_TPI =7.01;
DGro_GAPDH =42.129;
DGro_PGK =-885.9547;
DGro_PGYM =5.9;
DGro_ENO =-4.53;
DGro_PYK =-934.3262;
DGro_LDH =-65.0199;
DGro_FD =-188.7311;
DGro_GLUT2 =0;
DGro_LACT =0;

Keq_GLK_cytoplasm = exp(-DGro_GLK/RT)/P(1)/P(2)*P(3)*P(4)/h_cytoplasm;
Keq_G6PASE_cytoplasm = exp(-DGro_G6PASE/RT)*P(1)/P(4)*P(6);
Keq_PGI_cytoplasm = exp(-DGro_PGI/RT)/P(4)*P(5);
Keq_PFK_cytoplasm = exp(-DGro_PFK/RT)/P(2)*P(3)/P(5)*P(7)/h_cytoplasm;
Keq_FBP1_cytoplasm = exp(-DGro_FBP1/RT)*P(5)*P(6)/P(7);
Keq_ALD_cytoplasm = exp(-DGro_ALD/RT)/P(7)*P(9)*P(10);
Keq_TPI_cytoplasm = exp(-DGro_TPI/RT)/P(9)*P(10);
Keq_GAPDH_cytoplasm = exp(-DGro_GAPDH/RT)/P(6)/P(10)/P(11)*P(12)*P(13)/h_cytoplasm;
Keq_PGK_cytoplasm = exp(-DGro_PGK/RT)*P(2)^2/P(3)^2/P(12)*P(14);
Keq_PGYM_cytoplasm = exp(-DGro_PGYM/RT)/P(14)*P(15);
Keq_ENO_cytoplasm = exp(-DGro_ENO/RT)/P(15)*P(16);
Keq_PYK_cytoplasm = exp(-DGro_PYK/RT)*P(2)^2/P(3)^2/P(16)*P(17)*h_cytoplasm;
Keq_LDH_cytoplasm = exp(-DGro_LDH/RT)*P(11)/P(13)/P(17)*P(18)*h_cytoplasm;
Keq_FD_cytoplasm = exp(-DGro_FD/RT)/P(1)*P(6);
Keq_GLUT2 = exp(-DGro_GLUT2/RT)*P(1)/P(19);
Keq_LACT = exp(-DGro_LACT/RT)/P(18)*P(20)*h_cytoplasm^1/h_extracellular^1;
```

## FLUX EQUATIONS

## GLK\_cytoplasm

```
a=ATP_cytoplasm;
b=glucose_cytoplasm;
p=glucose6phos_cytoplasm;
q=ADP_cytoplasm;
Vmax_GLK=0.0252;

KmGLC=7.5;
KmATP=0.26;
n=1.6;
ngkrp=2;
Kglc_gkrp=15;
bgkrp=0.7;
Kf6p_gkrp=0.010;
GLKfree=b^ngkrp/(b^ngkrp+(Kglc_gkrp)^ngkrp)*(1-bgkrp*fructose6phos_cytoplasm/(fructose6phos_cytoplasm+Kf6p_gkrp));
J_GLK_cytoplasm=(Vmax_GLK*GLKfree*a/(KmATP+a)*b^n/(b^n+(KmGLC)^n));
```

## G6PASE\_cytoplasm

```
a=glucose6phos_cytoplasm;
p=glucose_cytoplasm;
Vmax_G6PASE=0.0189;
Km_G6PASE=2;
J_G6PASE_cytoplasm=Vmax_G6PASE*a/(Km_G6PASE+a);
```

## PGI\_cytoplasm

```
a=glucose6phos_cytoplasm;
b=fructose6phos_cytoplasm;
Keq_PGI_cytoplasm_PGI=0.5157;
Km_GL6P=0.182;
Km_FR6P=0.071;
Vmax_PGI=0.42;
J_PGI_cytoplasm=Vmax_PGI/Km_GL6P*(a-b/Keq_PGI_cytoplasm)/(1+a/Km_GL6P+b/Km_FR6P);
```

## PFK\_cytoplasm

```
a=ATP_cytoplasm;
b=fructose6phos_cytoplasm;
p=fructose26phos_cytoplasm;
Vmax_PFK=0.007182;
Kmf6p=0.077;
Kif6p=0.012;
KmATP=0.111;
Kaf26p=0.001;
J_PFK_cytoplasm=Vmax_PFK*(1+p/Kaf26p)*(b*a/(Kif6p*KmATP+Kmf6p*a+KmATP*b+a*b));
```

## FBP1\_cytoplasm

```
a=fructose16phos_cytoplasm;
p=fructose6phos_cytoplasm;
q=fructose26phos_cytoplasm;
Vmax_FBP1=0.0042;
Kif26p=0.001;
Kmf16p=0.0013;
J_FBP1_cytoplasm=Vmax_FBP1/(1+q/Kif26p)*(a/(a+Kmf16p))+kfbp1*a;
```

## ALD\_cytoplasm

```
a=fructose16phos_cytoplasm;
q=dihydroxyacetonephos_cytoplasm;
p=glyceraldehydephos_cytoplasm;
Vmax_ALD=0.042;
Keq_ALD_cytoplasm_ALD=9.76e-2;
Kmf16p=0.0071;
Kmdhap=0.0364;
Kmgrap=0.0071;
Ki1grap=0.0572;
Ki2grap=0.176;
J_ALD_cytoplasm=Vmax_ALD/Kmf16p*(a-p*q/Keq_ALD_cytoplasm_ALD)/(1+a/Kmf16p+p/Ki1grap+q*(p+Kmgrap)/(Kmdhap*Ki1grap)+a*p/(Kmf16p*Ki2grap));
```

## TPI\_cytoplasm

```
p=glyceraldehydephos_cytoplasm;
a=dihydroxyacetonephos_cytoplasm;
Vmax_TPI=0.42;
Keq_TPI_cytoplasm_TPI=0.0545;
KmDHAP=0.59;
KmGRAP=0.42;
J_TPI_cytoplasm=Vmax_TPI/KmDHAP*(a-p/Keq_TPI_cytoplasm_TPI/1+a/KmDHAP+p/KmGRAP);
```

## GAPDH\_cytoplasm

```
a=glyceraldehydephos_cytoplasm;
b=Pi_cytoplasm;
c=NAD_cytoplasm;
p=bpg_cytoplasm;
q=NADH_cytoplasm;
Vmax_GAPDH=0.42;
Keq_GAPDH_cytoplasm_GAPDH=0.0868;
Knad=0.05;
Kgrap=0.005;
Kp=3.9;
Knadh=0.0083;
Kbpg13=0.0035;
J_GAPDH_cytoplasm=Vmax_GAPDH/(Knad*Kgrap*Kp)*(c*a*b-p*q/Keq_GAPDH_cytoplasm_GAPDH)/((1+c/Knad)*(1+a/Kgrap)*(1+b/Kp)+(1+q/Knadh)*(1+p/Kbpg13)-1);
```

## PGK\_cytoplasm

```
a=bpg_cytoplasm;
b=ADP_cytoplasm;
p=ATP_cytoplasm;
q=pg3_cytoplasm;
Vmax_PGK=0.4;
Keq_PGK_cytoplasm_PGK=7;
Kadp=0.35;
Kbpg13=0.002;
Katp=0.48;
Kpg3=1.2;
J_PGK_cytoplasm=Vmax_PGK/(Kadp*Kbpg13)*(b*a-p*q/Keq_PGK_cytoplasm_PGK)/((1+b/Kadp)*(1+p/Katp)+(1+p/Katp)*(1+q/Kpg3)-1);
```

## PGYM\_cytoplasm

```
a=pg3_cytoplasm;
p=pg2_cytoplasm;
Vmax_PGYM=0.42;
Keq_PGYM_cytoplasm_PGYM=0.1814;
Kpg3=5;
Kpg2=1;
J_PGYM_cytoplasm=Vmax_PGYM*(a-p/Keq_PGYM_cytoplasm_PGYM)/(a+Kpg3*(1+p/Kpg2));
```

## ENO\_cytoplasm

```
a=pg2_cytoplasm;
p=pep_cytoplasm;
Vmax_ENO=0.035994;
Keq_ENO_cytoplasm_ENO=0.0545;
Kpg2=1;
Kpep=1;
J_ENO_cytoplasm=Vmax_ENO*(a-p/Keq_ENO_cytoplasm)/(a+Kpg2*(1+p/Kpep));
```

## PYK\_cytoplasm

```
a=pep_cytoplasm;
b=ADP_cytoplasm;
p=ATP_cytoplasm;
q=fructose16phos_cytoplasm;
Vmax_PK=0.0462;
npep=3.5;
nfbp=1.8;
Kpep_dp=0.58;
Kpep_p=1.10;
Kpep_min=0.08;
a_dp=1;
a_p=1.1;
a_end=1;
Kfbp_dp=0.16;
Kfbp_p=0.35;
a_base_dp=0.08;
a_base_p=0.04;
Kadp=2.3;
gammaPK=0;
fdp=q^nfbp/(q^nfbp+(Kfbp_dp)^nfbp);
fp=q^nfbp/(q^nfbp+(Kfbp_p)^nfbp);
a_in_dp=(1-fdp)*(a_dp-a_end)+a_end;
a_in_p=(1-fp)*(a_p-a_end)+a_end;
Kpep_inp_dp=(1-fdp)*(Kpep_dp-Kpep_min)+Kpep_min;
Kpep_inp_p=(1-fp)*(Kpep_p-Kpep_min)+Kpep_min;
V23_dp=Vmax_PK*a_in_dp*(a^npep/(a^npep+(Kpep_inp_dp)^npep))*(b/(b+Kadp))*(a_base_dp+(1-a_base_dp)*fdp);
V23_p=Vmax_PK*a_in_p*(a^npep/(a^npep+(Kpep_inp_p)^npep))*(b/(b+Kadp))*(a_base_p+(1-a_base_p)*fdp);
J_PYK_cytoplasm=(1-gammaPK)*V23_dp+gammaPK*V23_p;
```

## LDH\_cytoplasm

```
a=pyruvate_cytoplasm;
b=NADH_cytoplasm;
p=lactate_cytoplasm;
q=NAD_cytoplasm;
Vmax_LDH=0.0126;
Keq_LDH_cytoplasm_LDH=1;

Km_pyr=0.495;
Km_lac=31.98;
Km_nad=0.984;
Km_nadh=0.027;
J_LDH_cytoplasm=Vmax_LDH/(Km_pyr*Km_nadh)*(a*b-p*q/Keq_LDH_cytoplasm_LDH)/((1+b/Km_nadh)*(1+a/Km_pyr)+(1+p/Km_lac)*(1+q/Km_nad)-1);
```

## FD\_cytoplasm

```
a=glucose_cytoplasm;
p=Pi_cytoplasm;
n=10;
km=9;
vmax=1;
J_FD_cytoplasm=(vmax*a^n)/(km^n+a^n);
```

## GLUT2:extracellular\_to\_cytoplasm

```
Vmax_GLUT2 = 0.42;
Km_GLUT2 = 42;
Keq_GLUT2_GLUT2 = 1;
J_GLUT2_extracellular_to_cytoplasm = Vmax_GLUT2 / Km_GLUT2 * (glucose_extracellular - glucose_cytoplasm/Keq_GLUT2_GLUT2)/ (1+glucose_extracellular/Km_GLUT2 + glucose_cytoplasm/Km_GLUT2);
```

## LACT:cytoplasm\_to\_extracellular

```
Vmax_LACT = 20.000000000000e-04;
Keq_LACT_LACT = 1.151708351469357e+02;
Km_LACT = 0.000000006938102;
J_LACT_cytoplasm_to_extracellular = Vmax_LACT/Km_LACT *(lactate_cytoplasm - lactate_extracellular/Keq_LACT_LACT) / (1 + lactate_cytoplasm / Km_LACT + lactate_extracellular/Km_LACT) ;
```

## REACTANT TIME DERIVATIVES

```
f(1,:) = ( 0  - 1*J_GLK_cytoplasm + 1*J_G6PASE_cytoplasm - 1*J_FD_cytoplasm + 1*J_GLUT2_extracellular_to_cytoplasm ) / VWater_cytoplasm; % glucose_cytoplasm
f(2,:) = ( 0  - 1*J_GLK_cytoplasm - 1*J_PFK_cytoplasm + 2*J_PGK_cytoplasm + 2*J_PYK_cytoplasm ) / VWater_cytoplasm; % ATP_cytoplasm
f(3,:) = ( 0  + 1*J_GLK_cytoplasm + 1*J_PFK_cytoplasm - 2*J_PGK_cytoplasm - 2*J_PYK_cytoplasm ) / VWater_cytoplasm; % ADP_cytoplasm
f(4,:) = ( 0  + 1*J_GLK_cytoplasm - 1*J_G6PASE_cytoplasm - 1*J_PGI_cytoplasm ) / VWater_cytoplasm; % glucose6phos_cytoplasm
f(5,:) = ( 0  + 1*J_PGI_cytoplasm - 1*J_PFK_cytoplasm + 1*J_FBP1_cytoplasm ) / VWater_cytoplasm; % fructose6phos_cytoplasm
f(6,:) = ( 0  ); % [clamped] % Pi_cytoplasm
f(7,:) = ( 0  + 1*J_PFK_cytoplasm - 1*J_FBP1_cytoplasm - 1*J_ALD_cytoplasm ) / VWater_cytoplasm; % fructose16phos_cytoplasm
f(8,:) = ( 0  ) / VWater_cytoplasm; % fructose26phos_cytoplasm
f(9,:) = ( 0  + 1*J_ALD_cytoplasm - 1*J_TPI_cytoplasm ) / VWater_cytoplasm; % dihydroxyacetonephos_cytoplasm
f(10,:) = ( 0  + 1*J_ALD_cytoplasm + 1*J_TPI_cytoplasm - 1*J_GAPDH_cytoplasm ) / VWater_cytoplasm; % glyceraldehydephos_cytoplasm
f(11,:) = ( 0  - 1*J_GAPDH_cytoplasm + 1*J_LDH_cytoplasm ) / VWater_cytoplasm; % NAD_cytoplasm
f(12,:) = ( 0  + 1*J_GAPDH_cytoplasm - 1*J_PGK_cytoplasm ) / VWater_cytoplasm; % bpg_cytoplasm
f(13,:) = ( 0  + 1*J_GAPDH_cytoplasm - 1*J_LDH_cytoplasm ) / VWater_cytoplasm; % NADH_cytoplasm
f(14,:) = ( 0  + 1*J_PGK_cytoplasm - 1*J_PGYM_cytoplasm ) / VWater_cytoplasm; % pg3_cytoplasm
f(15,:) = ( 0  + 1*J_PGYM_cytoplasm - 1*J_ENO_cytoplasm ) / VWater_cytoplasm; % pg2_cytoplasm
f(16,:) = ( 0  + 1*J_ENO_cytoplasm - 1*J_PYK_cytoplasm ) / VWater_cytoplasm; % pep_cytoplasm
f(17,:) = ( 0  + 1*J_PYK_cytoplasm - 1*J_LDH_cytoplasm  ) / VWater_cytoplasm; % pyruvate_cytoplasm
f(18,:) = ( 0  + 1*J_LDH_cytoplasm - 1*J_LACT_cytoplasm_to_extracellular/VRegion_cytoplasm*VRegion_extracellular  ) / VWater_cytoplasm; % lactate_cytoplasm
f(19,:) = ( 0  - 1*J_GLUT2_extracellular_to_cytoplasm/VRegion_extracellular*VRegion_cytoplasm ) / VWater_extracellular; % Glucose extracellular
f(20,:) = ( 0  + 1*J_LACT_cytoplasm_to_extracellular ) / VWater_extracellular; % lactate_extracellular
```

## ION EQUATIONS

COMPARTMENT cytoplasm:

```
ii = [1   2   3   4   5   6   7   8   9  10  11  12  13  14  15  16  17  18]; % Indices of SVs in compartment cytoplasm
% PARTIAL DERIVATIVES
pHBpM = -sum( (h_cytoplasm*x(ii)'./Kh(ii))./(Km(ii).*P(ii).^2) );
pHBpK = -sum( (h_cytoplasm*x(ii)'./Kh(ii))./(Kk(ii).*P(ii).^2) );
pHBpH = +sum( (1+m_cytoplasm./Km(ii)+k_cytoplasm./Kk(ii)).*x(ii)'./(Kh(ii).*P(ii).^2) );
pMBpH = -sum( (m_cytoplasm*x(ii)'./Km(ii))./(Kh(ii).*P(ii).^2) );
pMBpK = -sum( (m_cytoplasm*x(ii)'./Km(ii))./(Kk(ii).*P(ii).^2) );
pMBpM = +sum( (1+h_cytoplasm./Kh(ii)+k_cytoplasm./Kk(ii)).*x(ii)'./(Km(ii).*P(ii).^2) );
pKBpH = -sum( (k_cytoplasm*x(ii)'./Kk(ii))./(Kh(ii).*P(ii).^2) );
pKBpM = -sum( (k_cytoplasm*x(ii)'./Kk(ii))./(Km(ii).*P(ii).^2) );
pKBpK = +sum( (1+h_cytoplasm./Kh(ii)+m_cytoplasm./Km(ii)).*x(ii)'./(Kk(ii).*P(ii).^2) );
% PHIs
J_H = (0 + 1*J_GLK_cytoplasm + 0*J_G6PASE_cytoplasm + 0*J_PGI_cytoplasm + 1*J_PFK_cytoplasm + 0*J_FBP1_cytoplasm + 0*J_ALD_cytoplasm + 0*J_TPI_cytoplasm + 1*J_GAPDH_cytoplasm + 0*J_PGK_cytoplasm + 0*J_PGYM_cytoplasm + 0*J_ENO_cytoplasm - 1*J_PYK_cytoplasm - 1*J_LDH_cytoplasm + 0*J_FD_cytoplasm - 1*J_LACT_cytoplasm_to_extracellular/VRegion_cytoplasm*VRegion_extracellular) / VWater_cytoplasm;
J_M = (0) / VWater_cytoplasm;
J_K = (0) / VWater_cytoplasm;
Phi_H = J_H - sum( h_cytoplasm*f(ii)'./(Kh(ii).*P(ii)) );
Phi_M = -sum( m_cytoplasm*f(ii)'./(Km(ii).*P(ii)) );
Phi_K = J_K -sum( k_cytoplasm*f(ii)'./(Kk(ii).*P(ii)) );
% ALPHAs
aH = 1 + pHBpH;
aM = 1 + pMBpM;
aK = 1 + pKBpK;
% ADDITIONAL BUFFER for [H+]
aH = 1 + pHBpH + BX(1)/K_BX(1)/(1+h_cytoplasm/K_BX(1))^2; % M
% DENOMINATOR
D = aH*pKBpM*pMBpK + aK*pHBpM*pMBpH + aM*pHBpK*pKBpH - ...
    aM*aK*aH - pHBpK*pKBpM*pMBpH - pHBpM*pMBpK*pKBpH;
% DERIVATIVES for H,Mg,K
% f(21,:) =  ( (pKBpM*pMBpK - aM*aK)*Phi_H + ...
%             (aK*pHBpM - pHBpK*pKBpM)*Phi_M + ...
%             (aM*pHBpK - pHBpM*pMBpK)*Phi_K ) / D;
        % DERIVATIVES for H,Mg,K
f(21,:) =  0;
f(22,:) =  0;
f(23,:) =  0;
% COMPARTMENT extracellular:
ii = [19  20]; % Indices of SVs in compartment extracellular
% PARTIAL DERIVATIVES
pHBpM = -sum( (h_extracellular*x(ii)'./Kh(ii))./(Km(ii).*P(ii).^2) );
pHBpK = -sum( (h_extracellular*x(ii)'./Kh(ii))./(Kk(ii).*P(ii).^2) );
pHBpH = +sum( (1+m_extracellular./Km(ii)+k_extracellular./Kk(ii)).*x(ii)'./(Kh(ii).*P(ii).^2) );
pMBpH = -sum( (m_extracellular*x(ii)'./Km(ii))./(Kh(ii).*P(ii).^2) );
pMBpK = -sum( (m_extracellular*x(ii)'./Km(ii))./(Kk(ii).*P(ii).^2) );
pMBpM = +sum( (1+h_extracellular./Kh(ii)+k_extracellular./Kk(ii)).*x(ii)'./(Km(ii).*P(ii).^2) );
pKBpH = -sum( (k_extracellular*x(ii)'./Kk(ii))./(Kh(ii).*P(ii).^2) );
pKBpM = -sum( (k_extracellular*x(ii)'./Kk(ii))./(Km(ii).*P(ii).^2) );
pKBpK = +sum( (1+h_extracellular./Kh(ii)+m_extracellular./Km(ii)).*x(ii)'./(Kk(ii).*P(ii).^2) );
% PHIs
J_H = (0 + 1*J_LACT_cytoplasm_to_extracellular) / VWater_extracellular;
J_M = (0) / VWater_extracellular;
J_K = (0) / VWater_extracellular;
Phi_H = J_H - sum( h_extracellular*f(ii)'./(Kh(ii).*P(ii)) );
Phi_M = -sum( m_extracellular*f(ii)'./(Km(ii).*P(ii)) );
Phi_K = J_K -sum( k_extracellular*f(ii)'./(Kk(ii).*P(ii)) );
% ALPHAs
aH = 1 + pHBpH;
aM = 1 + pMBpM;
aK = 1 + pKBpK;
% ADDITIONAL BUFFER for [H+]
aH = 1 + pHBpH + BX(2)/K_BX(2)/(1+h_extracellular/K_BX(2))^2; % M
% DENOMINATOR
D = aH*pKBpM*pMBpK + aK*pHBpM*pMBpH + aM*pHBpK*pKBpH - ...
    aM*aK*aH - pHBpK*pKBpM*pMBpH - pHBpM*pMBpK*pKBpH;
% DERIVATIVES for H,Mg,K
% f(24,:) =  0;
f(24,:) =  ( (pKBpM*pMBpK - aM*aK)*Phi_H + ...
            (aK*pHBpM - pHBpK*pKBpM)*Phi_M + ...
            (aM*pHBpK - pHBpM*pMBpK)*Phi_K ) / D;
f(25,:) =  0;
f(26,:) =  0;
```

## ELECTROPHYS EQUATIONS

C\_extracellular\_to\_cytoplasm = par(1);

```
C_extracellular_to_cytoplasm = 10;
f(27) = ( 0) / C_extracellular_to_cytoplasm;
```

## FLUX VECTOR:

```
J = [ J_GLK_cytoplasm J_G6PASE_cytoplasm J_PGI_cytoplasm J_PFK_cytoplasm J_FBP1_cytoplasm J_ALD_cytoplasm J_TPI_cytoplasm J_GAPDH_cytoplasm J_PGK_cytoplasm J_PGYM_cytoplasm J_ENO_cytoplasm J_PYK_cytoplasm J_LDH_cytoplasm J_FD_cytoplasm J_GLUT2_extracellular_to_cytoplasm J_LACT_cytoplasm_to_extracellular];
```

Published with MATLAB® R2017a
