# Supplementary material for: Modelling the impact of changes in the extracellular environment on the cytosolic free NAD+/NADH ratio during cell culture
Source: PLoS One. 2018 Nov 29;13(11):e0207803. doi: 10.1371/journal.pone.0207803 (PMC6264472; doi:10.1371/journal.pone.0207803)
Supplement: S2 File — Zip file containing html versions of model code. (ZIP) [file pone.0207803.s004.zip › html/HGM.html]

HEPATOCARCINOMA GLYCOLYSIS MODEL 

# HEPATOCARCINOMA GLYCOLYSIS MODEL

```
author: Ross Kelly
        Department of Applied Mathematics
        Liverpool John Moores University
        R.A.Kelly@ljmu.ac.uk
date:   23.08.2018
```

## Contents

- Liver hepatic glucose metabolism model RK18
- Figure 9 Simulation
- Plot results

## Liver hepatic glucose metabolism model RK18

```
% Load initial model setup
load setup.mat
options = odeset('MaxStep',5e-2, 'RelTol',1e-4,'AbsTol',1e-10);

% Load initial conditions
load x0b.mat
x0 = x0b;
```

## Figure 9 Simulation

```
disp( 'Recreating Figure 9 ...' );
kfbp1 = 8e-3;
t = [];
y=[];

% Simulate to steady state
tspan =linspace(0,10000);

x0(20) = 0;
x0(19) = 5;
[t,y] = ode15s(@dXdT_HGM, tspan, x0, options,T, BX, K_BX,params,kfbp1);

x0 = y(end,:);
x01 = x0;
% Simulate for unchanged extracellular substrates
tspan = linspace(0,120);
[tt,yy] = ode15s(@dXdT_HGM, tspan, x0, options,T, BX, K_BX,params,kfbp1);

for i = 1:length(tt)
    [junk,J(i,:)] = dXdT_HGM( tt(i), yy(i,:).', T,BX, K_BX,params,kfbp1);
end

x = yy;
torig = tt;
NADHbasal = x(:,11);
NADbasal = x(:,13);
GAPDHbasal = J(:,modelInfo.FluxID.GAPDH_cytoplasm);
LDHbasal = J(:,modelInfo.FluxID.LDH_cytoplasm);

% Perturb extracellular glucose
glc = (1:1:25);
NAD = [];
NADH = [];
LDH = [];
GAPDH = [];

for i = 1:length(glc)
    x0(19) = glc(i);

tspan =linspace(0,120);

[t1,y1] = ode15s(@dXdT_HGM, tspan, x0, options,T, BX, K_BX,params,kfbp1);

for i = 1:length(t1)
    [junk,J(i,:)] = dXdT_HGM( t1(i), y1(i,:).', T,BX, K_BX,params,kfbp1);
end

x = y1;
torig = t;

nad1 = x(:,11);
NAD = [NAD nad1];

nadh1 = x(:,13);
NADH = [NADH nadh1];

ldh = (J(:,modelInfo.FluxID.LDH_cytoplasm));
LDH = [LDH ldh];

gapdh = (J(:,modelInfo.FluxID.GAPDH_cytoplasm));
GAPDH = [GAPDH gapdh];

end

% Simulate extracellular lact
kfbp1 = 8e-3;
t = [];
y=[];

lac = (0:1:40);
NAD2 = [];
NADH2 = [];
LDH2 = [];
GAPDH2 = [];

% Perturb extracellular lactate
for i = 1:length(lac)
    x01(20) = lac(i);

tspan =linspace(0,120);


[t2,y2] = ode15s(@dXdT_HGM, tspan, x01, options,T, BX, K_BX,params,kfbp1);

for i = 1:length(t2)
    [junk,J2(i,:)] = dXdT_HGM( t2(i), y2(i,:).', T,BX, K_BX,params,kfbp1);
end

x = y2;
torig = t;

nad2 = x(:,11);
NAD2 = [NAD2 nad2];

nadh2 = x(:,13);
NADH2 = [NADH2 nadh2];

ldh2 = (J2(:,modelInfo.FluxID.LDH_cytoplasm));
LDH2 = [LDH2 ldh2];

gapdh2 = (J2(:,modelInfo.FluxID.GAPDH_cytoplasm));
GAPDH2 = [GAPDH2 gapdh2];

end
```

## Plot results

```
run Plot_Fig9.m
```

Published with MATLAB® R2017a
