# Supplementary material for: Modelling the impact of changes in the extracellular environment on the cytosolic free NAD+/NADH ratio during cell culture
Source: PLoS One. 2018 Nov 29;13(11):e0207803. doi: 10.1371/journal.pone.0207803 (PMC6264472; doi:10.1371/journal.pone.0207803)
Supplement: S2 File — Zip file containing html versions of model code. (ZIP) [file pone.0207803.s004.zip › html/Plot_Fig9.html]

Plot\_Fig9 

## Contents

- HEPATOCARCINOMA GLYCOLYSIS MODEL PLOT FILE
- Fig 9

## HEPATOCARCINOMA GLYCOLYSIS MODEL PLOT FILE

```
author: Ross Kelly
        Department of Applied Mathematics
        Liverpool John Moores University
        R.A.Kelly@ljmu.ac.uk
date:   23.08.2018
```

```
set(0,'defaulttextfontsize',28)
set(0,'defaulttextfontname','Times New Roman')
set(0,'defaultaxesfontsize',28)
set(0,'defaultaxesfontname','Times New Roman')
```

## Fig 9

```
    cc=jet(25);
    set(0,'DefaultAxesColorOrder',cc)

    GAPDHa = GAPDH./GAPDHbasal*100-100;
    LDHa = LDH./LDHbasal*100-100;

    figure(2),clf
    subplot(2,2,1)
    plot(t1,GAPDHa)
    xlabel('Time (min)')
    ylabel('GAPDH Flux (mM min^-^1)')
    axis square
    c = colorbar;
    ticks = linspace(0,25,6)';
    Ticks = num2str(ticks);
    c.TickLabels = Ticks;
    c.Label.String = 'Extracellular Glucose [mM]';
    ax = gca;
    ax.FontSize = 22;

    subplot(2,2,2)
    plot(t1,LDHa)
    xlabel('Time (min)')
    ylabel('LDH Flux (mM min^-^1)')
    axis square
    c = colorbar;
    ticks = linspace(0,25,6)';
    Ticks = num2str(ticks);
    c.TickLabels = Ticks;
    c.Label.String = 'Extracellular Glucose [mM]';
    ax = gca;
    ax.FontSize = 22;


    cc=jet(41);
    set(0,'DefaultAxesColorOrder',cc)

    GAPDHa = GAPDH2./GAPDHbasal*100-100;
    LDHa = LDH2./LDHbasal*100-100;

    subplot(2,2,3)
    plot(t2,GAPDHa)
    xlabel('Time (min)')
    ylabel('GAPDH Flux (mM min^-^1)')
    axis square
    c = colorbar;
    ticks = linspace(0,40,6)';
    Ticks = num2str(ticks);
    c.TickLabels = Ticks;
    c.Label.String = 'Extracellular Lactate [mM]';
    c.Direction = 'Reverse';
    ax = gca;
    ax.FontSize = 22;

    subplot(2,2,4)
    plot(t2,LDHa)
    xlabel('Time (min)')
    ylabel('LDH Flux (mM min^-^1)')
    axis square
    c = colorbar;
    ticks = linspace(0,40,6)';
    Ticks = num2str(ticks);
    c.TickLabels = Ticks;
    c.Label.String = 'Extracellular Lactate [mM]';
    c.Direction = 'Reverse';
    ax = gca;
    ax.FontSize = 22;
```

Published with MATLAB® R2017a
